# Emerging Roles of the Nervous System in Gastrointestinal Cancer Development

**DOI:** 10.3390/cancers14153722

**Published:** 2022-07-30

**Authors:** Chunhua Wan, Xiaoqin Yan, Baoying Hu, Xinhua Zhang

**Affiliations:** 1 Department of Nutrition and Food Hygiene, School of Public Health, Nantong University, Nantong 226001, China; 2 Medical Insurance Management Office, Shanghai Pudong Hospital, Fudan University Pudong Medical Center, 2800 Gongwei Road, Pudong, Shanghai 201399, China; yanxiaoqin0104@126.com; 3 Department of Immunology, School of Medicine, Nantong University, Nantong 226001, China; huby86@ntu.edu.cn; 4 Department of Human Anatomy, Institute of Neurobiology, Medical School of Nantong University, Nantong 226001, China; 5 Co-Innovation Center of Neuroregeneration, Nantong University, Nantong 226001, China; 6 Key Laboratory of Neuroregeneration of Jiangsu Province and Ministry of Education, Nantong University, Nantong 226001, China

**Keywords:** nervous system, gastrointestinal cancer, chronic stress, neurogenesis, neurotransmitter, tumor microenvironment

## Abstract

**Simple Summary:**

Nerve–cancer cross-talk has increasingly become a focus of the oncology field, particularly in gastrointestinal (GI) cancers. The indispensable roles of the nervous system in GI tumorigenesis and malignancy have been dissected by epidemiological, experimental animal and mechanistic data. Herein, we review and integrate recent discoveries linking the nervous system to GI cancer initiation and progression, and focus on the molecular mechanisms by which nerves and neural receptor pathways drive GI malignancy.

**Abstract:**

Our understanding of the fascinating connection between nervous system and gastrointestinal (GI) tumorigenesis has expanded greatly in recent years. Recent studies revealed that neurogenesis plays an active part in GI tumor initiation and progression. Tumor-driven neurogenesis, as well as neurite outgrowth of the pre-existing peripheral nervous system (PNS), may fuel GI tumor progression via facilitating cancer cell proliferation, chemoresistance, invasion and immune escape. Neurotransmitters and neuropeptides drive the activation of various oncogenic pathways downstream of neural receptors within cancer cells, underscoring the importance of neural signaling pathways in GI tumor malignancy. In addition, neural infiltration also plays an integral role in tumor microenvironments, and contributes to an environment in favor of tumor angiogenesis, immune evasion and invasion. Blockade of tumor innervation via denervation or pharmacological agents may serve as a promising therapeutic strategy against GI tumors. In this review, we summarize recent findings linking the nervous system to GI tumor progression, set the spotlight on the molecular mechanisms by which neural signaling fuels cancer aggressiveness, and highlight the importance of targeting neural mechanisms in GI tumor therapy.

## 1. Introduction

Gastrointestinal (GI) cancers, composed of esophageal cancer (EC), gastric cancer (GC), pancreatic cancer (PC), colorectal cancer (CRC), and liver cancer (LC), are some of the most frequently-diagnosed cancers worldwide. GI tumors are estimated to account for over one quarter of all cancer cases and one third of cancer-associated deaths [1]. The development of these tumors is heavily influenced both by genetic predispositions and microenvironmental factors. While the genetic basis of GI cancer development has been intensively investigated, some microenvironmental grounds remain to be untangled [2].

Studies in recent years uncovered a close relationship between nerves and cancer development, especially in GI tumors [3]. Epidemiological data have provided solid evidence linking psychological factors, such as chronic stress and depression, to increased GI cancer risk in the general population [4]. Nevertheless, mechanistic knowledge of the cross-talk between nerve and GI cancers lags well behind other aspects of tumor biology, such as cancer genetics, immunity and metabolism. This is partially attributable to the complexity of nerve–cancer cross-talk, and the difficulty in establishing reliable experimental models to recapture their interactions [5,6]. For instance, while tumor-associated neurogenesis has been observed since several decades ago, the underlying mechanisms have only been partially elucidated in recent years [3]. During GI tumorigenesis, delicate coordination among different cell types and tumor microenvironment underlies active neurogenesis, whose process is technically intractable using in vitro models [7,8]. Likewise, the roles of nerve fibers in GI cancer malignancy involve various neurotransmitters, neurotrophins and neuropeptides [9]. Their source and impacts on tumor malignancy and microenvironment involve complicated molecular mechanisms, whose elucidation requires vigorous investigations by both basic and clinical researchers. As a consequence, the involvement of the nervous system in GI tumor development remains elusive and needs to be fully understood.

The role of nervous system in tumorigenesis involves a wide range of aspects, at both systemic (neuroendocrine system) and tissue-specific (via tumor-associated nerve fibers) levels [10]. While the role of the neuroendocrine pathway in tumorigenesis has been well-recognized, it is only very recently that tumor-associated neurogenesis has been established as an important driver of GI tumorigenesis [3]. Moreover, mounting data indicated that a variety of neurotransmitter, neurotrophin and neuropeptide receptors are widely expressed in GI tumors. These findings, combined with recent in vitro results showing that nerve fibers fuel solid tumor growth and malignancy, point to a direct involvement of neural infiltration in the development of GI cancers [8]. Neural infiltration and neurotransmitter pathways appear to affect various aspects of GI development and malignancy, such as growth, invasion, chemoresistance, immune evasion and tumor microenvironments (TME). More importantly, these discoveries may lead to novel therapeutic strategies against GI tumors and benefit patients with diminishing responses to current therapies. In this review, we will discuss recent advances in neurological aspects of GI tumorigenesis and present a brief summary of the involvement of nerve fiber outgrowth and neural receptor pathways in GI cancer chemoresistance, malignancy and metastasis. This review will also discuss the potential of targeting neurological pathways as new treatments of GI tumors.

## 2. Chronic Stress Contributes to GI Tumor Risk

GI tissues are heavily innervated by the enteric nervous system (ENS) [11]. The ENS is the intrinsic nervous system controlling the function of the gastrointestinal tract and other digestive organs [12,13]. Within the GI tract, the ENS consists of thousands of ganglia identified between the longitudinal and the circular muscle layers (myenteric plexuses) and between the muscle and mucosal layers (submucosal plexuses), the neural connections that communicate these ganglia and nerve fibers that innervate effector tissues [14]. The nerve fibers of the ENS are distributed extensively within all layers of GI tract, including muscle layers, the submucosa, mucosal crypts and epithelium, to facilitate a wide range of GI functions [15]. While the ENS can function autonomously ex vivo, normal innervation of the digestive system requires integrated communications among the ENS and other nervous systems, particularly the central nervous system (CNS). The bidirectional communication between the ENS and CNS, termed as gut–brain axis (GBA), plays a central role in governing GI function [12,13]. GBA also establishes a link between psychological activity and GI homeostasis, given the fact that mental conditions have solid implications in GI disease progression, and vice versa [16]. Chronic stress conditions, such as depression and anxiety, are established risk factors for various GI disorders, underscoring the importance of psychological factors in GI disease progression [17]. Chronic stress has been linked to the initiation and progression of chronic GI disorders, possibly through aberrant release of neurotransmitters and neuropeptides by the ENS [18].

While the role of psychological factors on GI tumorigenesis remains under debate and inconclusive, some epidemiological data indicated that traumatic events, such as loss of close relatives, may increase the risk of multiple GI tumors, including pancreatic, gastric and colorectal cancers [19,20]. In addition, moderate associations among other stress-related syndromes, such as depression and anxiety, and GI cancer incidence have been revealed [4,21]. These effects of psychological factors were perfectly captured by elegant animal studies showing that enriched physical environments attenuate colon and pancreatic cancer progression [22,23]. Importantly, clinical data and animal studies confirmed that chronic stress may facilitate precancerous lesions in the upper gastrointestinal tract [24,25]. Collectively, these results point to a potential link between chronic stress and increased GI cancer incident, highlighting the importance of psychological factors in the initiation of GI tumors.

**Figure 1:**
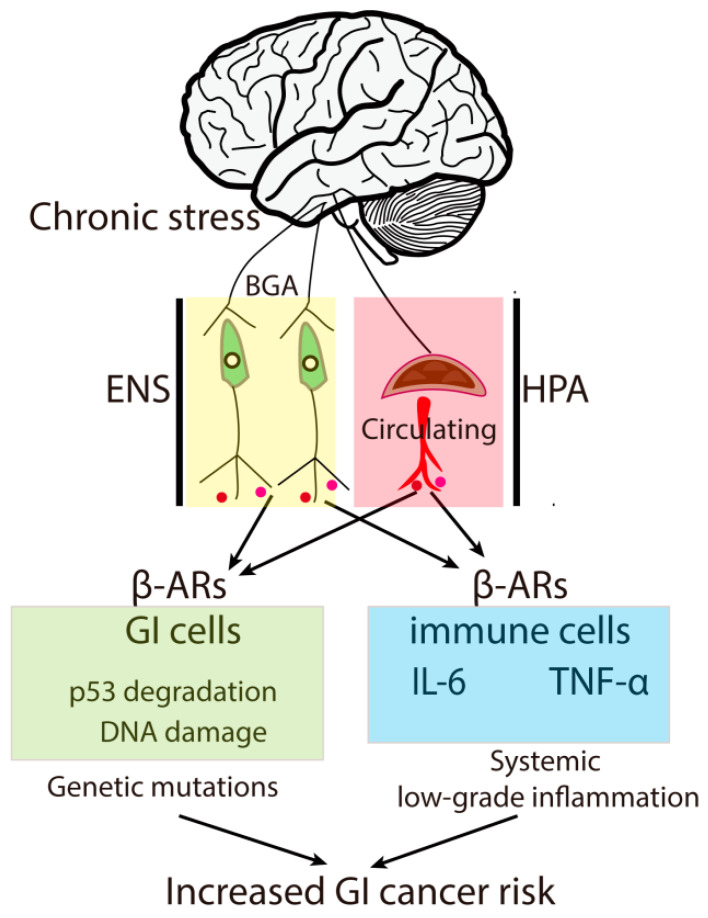
Schematic diagram showigng the molecular mechanisms by which chronic stress increases GI cancer risk. Chronic stress may facilitate β-AR signaling pathways via the ENS and HPA axis, leading to p53 degradation and DNA damage in GI somatic cells and the secretion of pro-inflammatory factors from immune cells. Through these mechanisms, chronic stress results in genetic mutations and systemic low-grade inflammation in GI tissues, causing increased GI cancer risks. GBA, gut–brain axis; ENS, enteric nervous system; HPA, hypothalamus–pituitary–adrenal; β-ARs, β-Adrenergic receptors.

The mechanisms by which chronic stress exacerbates GI cancer risk are complex. Studies proposed that the neuroendocrine pathways, including the GBA and the hypothalamus–pituitary–adrenal (HPA) axis, may contribute to chronic stress-augmented GI cancer risk. This effect may involve local immune malfunction and inflammation via increased levels of stress hormones, such as cortisol, adrenaline and noradrenaline [26,27]. Early studies indicated that psychological factors are associated with increased levels of pro-inflammatory cytokines, such as IL-6, and systemic low-grade inflammation in human populations [28,29,30,31]. Animal studies confirmed that chronic stress induces low-grade inflammation and the upregulation of IL-6, TNF-α, and CRP, which may contribute to tumor initiation [32]. In addition to a role in promoting inflammation, recent studies provided other explanations linking chronic stress to GI cancer risk. For instance, it was recently revealed that chronic stress may accelerate the degradation of p53 via the β2-adrenoreceptor (β2-AR)/β-arrestin-1 pathway, leading to genomic instability and the accumulation of DNA damages [33]. Another study showed that glucocorticoids may decrease p53 protein level via SGK1-Mdm2 pathway, which augments ionizing radiation (IR)-induced tumorigenesis [34]. These data surprisingly implicated a mutagenic effect of chronic stress via stress hormone pathways. Together, these findings implicated an involvement of inflammation and genetic damage in chronic stress-induced GI tumor risk (Figure 1).

## 3. GI Tumorigenesis Initiates Active Neurogenesis and Neural Infiltration

While nerve fibers were observed within rectum cancer and other solid tumors nearly a century ago, the role of nerves in tumor progression has long received insufficient attention [35,36]. To date, neurogenesis and nerve fiber outgrowth have been characterized within major GI tumors [37,38,39,40]. GI tumors, such as pancreatic cancer, are among those with the highest prevalence of neural infiltration [3]. However, the origin of tumor-associated nerve fibers has been mysterious for a long period of time. Given the facts that the growth of neurons and nerve fibers are mostly developmentally related and that adult neurogenesis only occurs in very restricted brain regions and rarely outside central nervous system, the molecular mechanisms driving neurogenesis and nerve fiber outgrowth in these tumors remain incompletely understood.

Data obtained from gastric and colorectal cancers revealed that neurons may be directly generated by the re-differentiation of cancer stem cells (CSCs) within tumors [41]. Using CSC-derived xenograft models, Ran Lu et al. showed that neural cells with human origin were identified in the ganglia close to and within xenografts. Importantly, blockade of neural differentiation attenuated gastric and colorectal tumor progression in the xenograft models. Another potential source of tumor-resident neurons is the circulating neural progenitors that dissociate from central nervous system [42]. Data suggested that neural progenitors may dissociate from the subventricular zone following the disruption of the blood–brain barrier, circulate in the circulatory system and penetrate into tumor tissues. These landmark discoveries revolutionized our understanding of the diversity and complexity of neurogenesis within GI tumors, underscoring the importance of active neurogenesis in GI tumorigenesis. It should be noted that neural infiltration is different from perineural invasion (PNI), which is also frequently observed in multiple GI cancers, particularly pancreatic ductal adenocarcinoma (PDAC) and gastric cancer, as PNI essentially represents a chemotactic effect by which tumor cells migrate toward and eventually invade pre-existing nerves [43,44].

**Figure 2:**
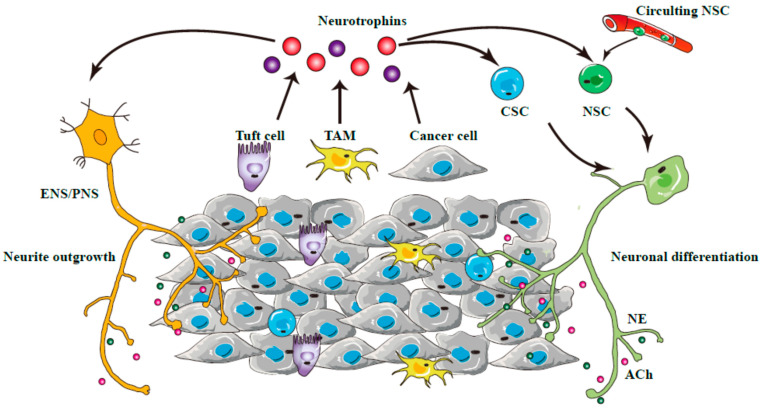
The mechanisms underlying neurogenesis during GI cancer development. The release of neurotrophins from various cell types, including tumor-associated macrophages (TAMs), tuft and cancer cells, may lead to neurite outgrowth of ENS/PNS and the differentiation of cancer stem cells (CSCs) and circulating neural stem cells (NSCs) into neurons, to fuel GI tumorigenesis. NE, Norepinephrine; ACh, acetylcholine.

It is noteworthy that multiple data have indicated that GI tumors may create a pro-neurogenic microenvironment to facilitate neural infiltration. One of the most obvious mechanisms is the release of a variety of neurotrophins, including nerve growth factor (NGF), brain-derived neurotrophic factor (BDNF) and glial cell-derived neurotrophic factor (GDNF), to facilitate neurogenesis [45,46]. A recent study showed that tuft cells, a type of chemosensory cells resided in intestinal epithelium, as well as nerves, may release acetylcholine to stimulate the production of NGF from gastric epithelial cells, leading to the growth of enteric nerves and accelerated gastric carcinogenesis [47]. Neurogenesis, similarly to angiogenesis, has been proposed to play an indispensable role in GI tumor progression and dissemination [48]. These findings implicated that tumor tissues may fuel neuronal differentiation, as well as neurite outgrowth from pre-existing ENS/PNS, by creating a neurogenic microenvironment [49,50]. Recent studies also indicated a role of immune cells in neural infiltration [51]. It was revealed that macrophages recruited by pancreatic cancer cells may secret GDNF to promote a cross-talk between nerve fibers and cancer cells [52]. Overall, these findings demonstrate neural infiltration as an integral part of GI tumor development (Figure 2).

## 4. Neural Infiltration Fuels GI Cancer Progression

The ENS plays a vital role in tissue homeostasis and metabolism of the gastrointestinal system. Enteric nerve fibers directly control lipid and glucose metabolism, and consequently the development of diabetes and fatty liver diseases [53,54]. However, the roles of nerve fibers in GI cancer development remain controversial and incompletely understood. Since neural infiltration has been identified across GI tumors, it is conceivable that the nervous system is involved in GI cancer progression. Indeed, nerve fiber infiltration has been linked to worsened prognosis in multiple GI tumors [55,56]. Accordingly, the density of tumoral nerve fibers may serve as indicative biomarkers for clinical outcomes of patients with GI tumors [55,57]. Daniel Albo et al. performed a quantitative analysis of nerve fibers in a colorectal cancer cohort of 236 patients and revealed that patients with high infiltration of PGP9.5-positive nerve fibers showed a 50% reduction in 5 year overall survival and disease-free survival, compared those with no detectable tumor-associated neurogenesis [37]. Furthermore, neural infiltration is also correlated with significantly higher GI tumor recurrence and worsened prognosis in patients with pancreatic cancer [58]. These data implicate a clinical relevance of the nervous system in GI tumors.

Though the nervous system has been linked to the development of all types of GI tumors, the degree of neural infiltration varies greatly among different GI tumors. For instance, nerve fibers have been observed in nearly all pancreatic tumors, 63% of colorectal cancer, 40% of gastric cancer and 38% of esophageal cancers [37,40,44,59]. However, S100-positive nerve fiber was absent within hepatocellular carcinoma (HCC) tumoral specimens, and can be only found in the capsule of a proportion of HCC tissues [60]. Accordingly, nerve fiber density is associated with worsened prognosis in the majority of GI tumors, but has no prognostic value in HCC [40,58,61,62]. These differences are very likely as a result of their histological patterns during the initiation of GI tumors. GI tract is a multi-layer tissue with neuronal bodies and nerve fibers as integral parts of the histological structure. Proximal connections between nerve fibers and various cell types imply an involvement of nerves in the initiation and progression of the tumors arising from GI tracts and pancreas [63,64]. On the other hand, the autonomic nervous system of the liver consists of branches of the splanchnic and vagal nerves. These nerves are mainly distributed around the portal vein, hepatic artery and bile duct, and have no direct connection with a majority of hepatocytes [65]. Nevertheless, neural signaling remains an important driver of hepatic carcinogenesis, despite the fact that nerve fibers have no prognostic merit. For instance, the expression of acetylcholinesterase, a key enzyme responsible for ACh breakdown, is inversely correlated with HCC growth and aggressiveness [66]. Another study showed that catecholamine neurotransmitter degrading enzyme monoamine oxidase A (MAOA) inhibits HCC metastasis via suppressing the adrenergic receptor signalling and the transactivation of EGFR pathway [67].

The mechanisms underpinning nerve-driven GI tumor progression are complex and involve various aspects of tumor aggressiveness. Some fascinating studies have shown that neural infiltration plays multifaceted roles in the malignancy of GI tumors [37,41]. However, because of the complexity of neural infiltration and the difficulty in establishing tumor innervation models in vitro, the precise mechanisms underlying the tumor-promoting roles of nerve fibers in GI cancers remain obscure. Nevertheless, since innervations typically work through the secretion of neurotransmitters and neuropeptides, the discovery of various neural receptors in tumor cells suggests that nerve fibers may directly fuel GI tumor malignancy via neural receptor pathways. Indeed, some delicate models of cancer-nerve cross talk have been established. By establishing an in vitro cross-talk model of murine sciatic nerves and cancer cells, several studies have shown that nerves may facilitate the proliferation, neural invasion and metastasis [68,69,70]. Notably, several signaling pathways, including GDNF-RET and SLIT2-ROBO, have been implicated in this cross-talk. Moreover, neural signals may also contribute to tumor resistance to chemotherapy. While clinical data supporting an association between nerve fiber density and tumor chemoresistance are lacking, mounting in vitro data have suggested critical involvement of assorted neurotransmitter pathways in the responsiveness to chemotherapeutic agents. Neurological pathways, such as β-adrenergic receptors (β-ARs) and acetylcholine receptors (AChRs), have been shown to promote chemoresistance of GI tumors via downstream oncogenic effectors [71,72,73]. In addition, neural signaling may promote the epithelial-mesenchymal transition (EMT) of GI tumors [74,75,76]. Combined, these findings implied that nerve fibers may facilitate the activation of conventional oncogenic signaling in GI tumors, and contribute to various aspects of tumor malignancies.

## 5. The Roles of Neurotransmitter, Neurotrophin and Neuropeptide Receptor Pathways in Gastrointestinal Cancer Development

Histological examinations have revealed that nerve fibers have been well characterized within and around GI tumors. Since tumor-associated nerves can be considered as part of peripheral nervous system, tumor innervation, via nerve fibers surrounding and within the tumor, is supposed to work in a mechanism similar to non-tumorous tissues, namely through the secretion of neuroactive chemicals and peptides from nerve terminals. Mostly, these molecules function locally via paracrine routes and act on their receptors that are distributed on the surface of tumor and stromal cells. As stated above, the expression of neural receptors on tumor cells may plays a key role in nerve fiber-primed GI tumor progression. Thus, the distribution and function of tumor-residual neural receptors are of great importance to determine the major pathways involved [77]. Dysregulated expression of assorted neural receptors, including neurotransmitter receptors and neuropeptide receptors, has been reported in GI tumors, and linked to the enhanced malignancy of tumor cells. These studies suggested that a variety of neural receptors are expressed across GI tumors, with many attracting particular attention. Below, we briefly discuss the roles of neural receptor pathways in GI tumorigenesis. In addition, we summarize the current literature indicating the expression, downstream effectors and function of neurotransmitter receptors (Table 1) in GI tumors.

### 5.1. β-Adrenergic Receptors

β-ARs are critical downstream effectors of neurotransmitters released from sympathetic nerve terminals. As members of G protein-coupled receptors (GPCRs), β-ARs may transmit intracellular signaling via G protein-mediated mechanisms and through adaptor proteins β-arrestin [78]. Studies revealed that all three members of β-ARs (β1-AR, β2-AR and β3-AR) are widely expressed across diverse tumor types [79]. Because of their importance in stress response and the sympathetic nervous system, β-ARs are among the most intensively investigated neurotransmitter receptor pathways in GI tumors [80,81]. β-ARs, particularly β2-AR, have been implicated in various malignant behaviors of GI tumors, including proliferation, chemoresistance and metastasis, using both elegant in vivo and in vitro models. Using a KC (LSL-Kras^+/G12D^; Pdx1-Cre) pancreatic cancer model, Bernhard Renz et al. [82] revealed that chronic restraint stress (CRS) facilitated β2-AR-dependent PDAC growth, NGF secretion and intratumoral neural density. Blockade of β2-AR and NGF/Trk pathways decreased cancer incidence and extended the survival of KPC (LSL-Kras^+/G12D^;LSL-Trp53^+/R172H^;Pdx1-Cre) mice, directly linking chronic stress to PDAC development via a β-AR/neurotrophinloop [82]. The tumor-facilitating effects of β-ARs have also been validated by selective β-AR antagonists, such as atenolol (β1-AR) and ICI 118, 551 (β2-AR) [83,84]. Mechanistic studies revealed that β-ARs have broad impacts on assorted classical oncogenic pathways, such as HIF-1α, AKT and ERK pathways [85,86]. Coinciding with these findings, our study showed that β2-adrenergic receptor (β2-AR) may facilitate the PCBP2-mediated translation of c-Myc to promote the proliferation of pancreatic cancer cells [87]. These data suggested that β-AR pathways are exploited by GI cancers to facilitate downstream oncogenic effectors, highlighting β-AR signaling as integral players of the oncogenic network in GI tumors.

### 5.2. Acetylcholine Receptors

Acetylcholine receptor (AChR) family proteins are composed of two subtypes, nicotinic (nAChR) and muscarinic (mAChR), both of which have been reportedly involved in GI tumor progression. nAChRs, such as α7nAChR, are of particular concern, partially because of their link to smoking-induced GI tumorigenesis via transmitting nicotine-mediated signals [88,89]. As ionotropic receptors, nAChRs may function as ligand-mediated ion channels, but also signal through intracellular signaling transducers [90]. Through these mechanisms, nAChRs may be coupled to various downstream signaling, such as JAK2/STAT3 and NF-κB signaling, leading to aggressive behaviors [91,92,93]. Different from nAChRs, mAChRs belong to metabotropic GPCRs that primarily transmit signals via G proteins and adenylyl cyclase. mAChRs consist of five members, termed m1–5 AChR, all of which are, to some extent, expressed in GI tract [94]. In this regard, m3AChR is a key subtype functionally associated with GI tumorigenesis, probably because of its preferential expression in stem cells [95]. Genetic ablation of m3AChR attenuates colon tumorigenesis, indicating a tumor-promoting function of this receptor [96]. In line with this finding, treatment with m3AChR agonist carbachol activates protein kinase cascades and the proliferation of gastric and colorectal tumor cells [97,98]. Interestingly, studies revealed that mAChR-mediated ERK signaling and cell proliferation may involve the transactivation of the EGFR pathway [99,100]. It should be noted that, while the AChR pathway was frequently associated with enhanced tumor aggressiveness, some studies also revealed tumor-suppressive functions of cholinergic signaling in GI tumors [101,102,103,104]. The inconsistence of these studies suggested that cholinergic signaling may function in a tumor context-dependent manner.

### 5.3. Glutamate Receptors (GluRs)

Serving as a principal neurotransmitter as well as a key metabolite, glutamate plays a unique role in GI tumor progression. Enrichment of glutamate, caused by aberrant metabolism of GI tumors, may trigger the activation of GluRs residing on the tumor cells [105,106]. A variety of GluRs, both ionotropic (iGluR) and metabotropic (mGluR), have been reportedly expressed in GI tumors [107]. Glutamate may promote the invasion of pancreatic cancer via ionotropic AMPA receptor-mediated Kras–MAPK signaling [108]. Multiple members of iGluR and mGluR have been implicated in the progression of colorectal cancer [109,110,111,112]. Likewise, NMDA receptor NR1 and NR2A subunits have been reportedly involved in the malignancy of gastric cancer [113,114]. Of great intrigue was Leanne Li et al.’s discovery that NMDAR is highly expressed in invasive fronts of pancreatic neuroendocrine tumorigenesis (PNET), and may drive tumor invasiveness through fluid flow-induced autocrine glutamate signaling circuit [115]. Collectively, these studies demonstrate the crucial roles of GluRs in GI neoplasia and underscore the importance of targeting glutamate metabolism and its receptor pathways as therapeutic strategies for GI tumors.

### 5.4. Dopamine (DA) and 5-HT (Serotonin) Receptors

Receptors 5-HT and DA are both abundantly produced in GI tract and play vital roles in GI homeostasis [116,117]. Accordingly, it is conceivable that these neurotransmitter pathways play critical roles in GI disease progression, including tumorigenesis. DA receptors (DARs) are composed of five class A GPCRs, and can be divided into D1-type (D1 and D5) and D2-type (D2, D3 and D4) subgroups [118]. Both of D1 and D2 types, to some degree, possess oncogenic properties in GI tumors. For instance, upregulation of DA and DA receptor D1 (DRD1) has been associated with tumor growth and invasion of hepatocellular carcinoma (HCC) [119]. Moreover, upregulated expression of DA receptor D2 (DRD2) is associated with elevated malignant potential of gastric cancer and PDAC, implicating a therapeutic merit of antagonizing DRD2 in these tumors [120,121]. Similar to DARs, serotonin receptor (5-HTR) pathways may also facilitate the progression of various GI tumors. Excessive production of serotonin, as a result of upregulated expression of serotonin biosynthesis rate-limiting enzyme tryptophan hydroxylase 1 (TPH1), contributes to NLRP3 inflammasome activation via serotonin receptor HTR3A and the acceleration of colorectal cancer development [122]. The accumulation of serotonin was observed in a Kras/p53-driven pancreatic cancer model, and linked to Warburg Effect and accelerated growth of pancreatic tumors [123]. An in vitro study also revealed that silencing the expression of 5-HT receptors 5-HT1B and 5-HT1D retarded the proliferation, clonogenicity and invasion of pancreatic cancer cells, pointing to a direct involvement of these receptor pathways in tumor cell malignancies [124].

**Table 1:** The expression and roles of neurotransmitter receptors in gastrointestinal cancer.

Receptors	Expression in Cancer/Mechanism	Downstream Effectors/Mechanisms	Effects	Reference
β-adrenergic receptors (β-ARs)				
β1-AR	Upregulated in EC	ERK, COX2	proliferation	[79,125]
	Upregulated in metastatic GC			[126]
	Expressed in PC	AKT, ERK, HIF-1α		[127]
	Upregulated in CRC			[79]
β2-AR	Upregulated in GC	STAT3, AP-1, MUC4	Proliferation, Chemoresistance, metastasis	[126,128,129]
	Upregulated in PC	HIF-1α, ERK, PCBP2, AKR1B1, CDC42	Proliferation, Angiogenesis, invasion	[83,85,87,127,130,131]
	Upregulated in CRC	EGFR-Akt/ERK	Proliferation, viability	[86,132]
	Upregulated in HCC	YB-1/β-catenin	metastasis	[76,133]
β3-AR	Upregulated in CRC			[134]
Acetylcholine receptors (AChRs)				
α3nAChR	Expressed in ESCC	YAP1	Proliferation, migration	[135]
α5nAChR	Expressed in GC	AKT	Chemoresistance	[136]
α7nAChR	Upregulated in EC	AKT/FOXO1/OTUD3/VEGF	lymphatic metastasis	[137]
	Expressed in GC	E-cadherin, ZEB-1, fibronectin, AKT, MCL-1, BCL-2	Migration, chemoresistance	[73,138,139]
	Upregulated in PC	MUC4, 29864419	Stemness, metastasis	[91,140]
	Expressed in CRC	NF-κB, Fibronectin, Snail, ZEB1	Migration	[141,142,143,144]
	Upregulated in CC	EMT, ERK	Proliferation, viability, migration	[74,145]
	Upregulated in HCC	TRAF6/NF-κB	Proliferation, chemoresistance	[72,93]
m1AChR	Expressed in HCC	EMT, PI3K/AKT	Invasion	[146]
m3AChR	Upregulated in GC	EGFR, AKT, ERK	Proliferation, viability	[100,147,148]
	Upregulated in PC			[149]
	Upregulated in CRC	Calcium, MMP7, EGFR, p38, ERK, AKT	Proliferation, viability	[150,151,152,153]
	Upregulated in CC		Proliferation, metastasis	[154]
Glutamate receptors (GluRs)				
AMPA receptor (GluR1–4)	Downregulated in PC	Kras-MAPK	invasion	[108]
	Downregulated in CRC (GluR4) /DNA methylation			[155]
NMDA receptors (NR1–3)	Upregulated in PC	AKT, ERK, CaMK II, HIF-1α	Proliferation, migration	[156,157]
	Expressed in GC		proliferation	[113]
	Upregulated in CRC (NR2D)	HIF-1α, AKT, ERK, CaMK II	migration, angiogenesis	[110,158]
	Upregulated in HCC			[159]
Kainate receptor	Downregulated in GC (GRIK2) /DNA methylation		Impaired Growth, migration	[160]
	Upregulated in GC (GRIK3)			[161]
metabotropic glutamate receptors (mGluRs)	Upregulated in PC (mGluR1)	PI3K/AKT/mTOR	Viability	[162,163]
	Upregulated in CRC (mGluR4)		5-FU resistance, recurrence	[109,164]
	Expressed in HCC (mGluR5)	Calcium, MAPK	Chemoresistance	[165]
Dopamine receptors (DRs)				
DRD1	Upregulated in ESCC			[166]
	Expressed in PC		Stemness, growth, migration	[167]
	Upregulated in HCC	cAMP/PI3K/AKT/CREB	Proliferation, metastasis	[119]
DRD2	Upregulated in ESCC		lymph node metastasis	[166]
	Upregulated in GC		Proliferation	[120]
	Upregulated in PC	Calcium, PKA	Proliferation, viability, migration	[121]
DRD5	Upregulated in EC	mTOR, AKT, Warburg effect	proliferation	[168]
	Expressed in GC	mTOR	Impaired growth, autophagy	[169]
	Expressed in CRC			[169]
	Upregulated in HCC	CD133, OCT4, and EpCam	Impaired growth, stemness, migration	[170]
Serotonin (5-HT) receptor				
5-HTRs	Expressed in CRC (5-HT1B, 5-HT3A, 5-HT3, and 5-HT4)	Calcium/CaMKIIα, NLRP3 inflammasome, MMP-12	Growth, angiogenesis, viability	[122,171,172,173,174]
	Expressed in PC (5-HT1B, 5-HT1D and 5-HT2B)	PI3K/AKT/mTOR, Warburg effect, uPAR/MMP-2, Integrin/Src/Fak	Growth, invasion	[123,124]
	Expressed in CC (5-HT1A, 5-HT2A, 5-HT2B, 5-HT4 and 5-HT6)		Growth	[175]
	Expressed in HCC (5-HT1B and 5-HT2B)	AKT, FOXO3a	Proliferation	[176]

EC, oesophageal cancer; ESCC, oesophageal squamous cell carcinoma; GC, gastric cancer; PC, pancreatic cancer; CRC, colorectal cancer; CC, cholangiocarcinoma; HCC, hepatocellular carcinoma.

### 5.5. Neurotrophin Receptors

While neurotrophin signals appear to promote neurogenesis during GI tumor initiation, neurotrophin receptors, termed tropomyosin-related kinases (TRKs), are also aberrantly upregulated in GI cancer cells [177]. The deregulation of neurotrophin receptors is frequently attributed to epigenetic mechanisms, such as DNA methylation [178,179,180]. Overexpression of NGF may promote the survival and motility of liver cancer cells via tropomyosin receptor kinase A (TrkA) pathway [181]. A similar effect of NGF-TrkA pathway in other GI tumors, such as colorectal and pancreatic cancers, has also been observed [182,183]. Likewise, BDNF-TrkB signaling fuels the growth and invasion of gastric and pancreatic carcinoma, and may serve as a therapeutic target against these malignancies [184,185]. Interestingly, whereas an earlier study reported a conditional tumor suppressive function of TrkC in colorectal cancer, cell culture and xenograft data indicated that TrkC may also elicit a tumor-promoting effect [180,186]. Similar to their functions in neurogenesis, TRK pathways may activate mitogenic pathways, such as AKT and Ras/MAPK, to drive the malignancy of GI tumors [187]. The expression and effects of neurotrophin receptors in GI tumors were summarized in Table 2.

### 5.6. Neuropeptide Receptors

Neuropeptides represent another important source of signal molecules bridging nerves and cancer cells. Given the fact that a variety of neuropeptides are abundantly produced in gastrointestinal tract and play key roles in the homeostasis of GI system, it is unsurprising that these neuropeptides are involved in GI tumorigenesis [216,217]. Many neuropeptides are secreted along with neurotransmitters from enteric nerve fibers, and serve as an additional layer of nerve–GI communication [218]. Notably, unlike neurotransmitters that typically play tumor-promoting roles, the roles of neuropeptides in GI tumors are complicated and controversial. An upregulated level of galanin has been linked to enhanced metastasis and chemoresistance of colorectal cancer [219,220]. Intriguingly, studies revealed that galanin may exert a tumor-suppressive effect in gastric and pancreatic carcinogenesis [221,222]. Likewise, methionine enkephalin (MENK) may function as tumor suppressor in gastric cancer development, but possesses oncogenic properties in some other GI tumors [223,224]. While neuropeptide Y (NPY) plays a critical role in the function of GI system, its role in GI tumorigenesis remains incompletely understood. Early studies suggested that the level of NPY was reduced in the serum of gastric and colorectal cancers [225]. However, recent experiments using genetically modified animals revealed that NPY may promote the proliferation of epithelial cell to facilitate DSS-induced intestinal carcinogenesis [226]. Overall, neuropeptides play complex and diverse roles in GI tumorigenesis. Much of this field remains incompletely understood and needs further investigations.

## 6. Neural Infiltration Is an Integral Part of GI Tumor Microenvironment (TME)

Because of the prevalence of nerve fiber infiltration in GI tumors, emerging evidence has indicated that they are an indispensable part of GI tumor microenvironment. Studies revealed that the nervous system is linked to aberrant function of tumor immune cells, fibroblasts and endothelial cells [77,227]. Neurotransmitters and other signal molecules secreted by nerve fibers have profound impacts on tumor environments and various types of stromal cells. Peripheral and tumor-associated nerves may foster a range of tumor-favoring microenvironments, exacerbating malignant characteristics of GI tumors.

### 6.1. Angiogenesis

Many neurotransmitter receptors such as β-ARs and ACh receptors (AChRs) have been reportedly expressed in vascular endothelial cells [228,229]. Chronic stress may facilitate VEGF expression and tumor angiogenesis through neurotransmitter signaling pathways [230]. Catecholamines, such as norepinephrine (NE) and epinephrine (E), have been well-recognized to promote tumor angiogenesis, through the secretion of VEGF [231,232]. On the other hand, DA pathway may inhibit angiogenesis in gastric cancer via DA receptor D2 (DRD2) on endothelial cells [233]. This effect of DRD2 may be attributed to enhanced endocytosis of VEGF receptor 2 (VEGFR2) [234].

### 6.2. Immune Regulation

Intensive studies in the past decades have demonstrated a fundamental role of the nervous system in immune regulation [235]. While the nervous system may fine-tune systemic inflammation and immune responses via direct innervations of lymphoid organs, nerve fibers within GI tumors play a vital role in the tumor immune microenvironment (TIME) via a paracrine mechanism. Neurotransmitters and neuropeptides secreted from local nerve terminals have strong influences on TIME through direct intervention on assorted immune cells. It has been well-documented that the receptors of various neurotransmitters, including catecholamines, GLU, 5-HT and ACh, are broadly expressed in diverse immune cells, including those infiltrating tumor tissues. For instance, functional β-ARs and α7nAChR have been reportedly expressed in tumor-associated macrophages (TAMs) in GI tumors [236,237]. Likewise, neural receptor pathways, including glutamate receptors and β-ARs, play indispensable roles in regulating tumor-eradicating activity of CD8^+^ cytotoxic T lymphocytes (CTLs) [238,239]. Aside from TAMs and CTLs, chronic stress has also been reported to regulate other immune cells, such as Treg and CD4 cells, via β-adrenergic receptor pathway in a pancreatic cancer model [240]. In agreement with these findings, studies based on an orthotopic PDAC model revealed that nerve fibers reprogram TIME and suppress intratumoral T-cell response to favor tumor progression [241]. Overall, in line with a tumor-promoting speculation of neural infiltration, most of these studies suggested that neural pathways may facilitate TIME in favor of tumor progression.

### 6.3. Chemotactic Effects

Studies in the past decades have indicated that neurotransmitters may serve as chemoattractants to guide cancer cell invasion towards nerve fibers. Perineural invasion has been observed in multiple GI tumors, which essentially represents a chemotactic effect of nerve fibers to tumor cells [242,243,244]. Using both in vivo and in vitro models, studies revealed a critical involvement of assorted neural pathways, such as substance P (SP)/NK-1R, NGF and β-adrenergic signals in pancreatic cancer PNI [193,245,246]. It is revealed that nerve fiber and neurotransmitters may increase the mobility of GI cancer cells in terms of inducing cytoskeleton remodeling and epithelial–mesenchymal transition [146,247]. In this regard, neural receptors may activate some key signals involved in cytoskeleton remodeling and cell migration, such as Kras–MAPK, MMPs and STAT3, and potentially drive directed migration of tumor cells along with neurotransmitter gradients [108,245].

## 7. Intervention of Neural Infiltration as Measurements of Cancer Treatment

Because of the critical role of neural infiltration in fueling GI tumor malignancy, it is unsurprising that intervention of nerve fibers may serve as a strategy to retard GI tumor progression. At present, multiple strategies have been proposed to block nerve–cancer cross-talks, among which denervation and pharmacological blockade of neural receptor pathways are some most well-characterized approaches.

Studies in the past decades have indicated promising effects of denervation in reducing cancer incidence and progression. Chun-Mei Zhao et al. reported that both surgical and pharmacological denervation reduced gastric tumor incidence, stemness and chemoresistance [248]. Similarly, denervation of the pancreas reduces cancer initiation and invasion in a Kras-driven PDAC model [249]. Data obtained from colorectal cancer also support the notion that denervation may be beneficial to the prevention of tumor incidence and progression [250,251]. However, it remains unclear whether denervation may prevent tumor-associated neurogenesis, or simply block the influence of the pre-existing peripheral nervous system on tumorigenesis.

Despite the fact that denervation exhibits considerable merit in preventing tumor progression, the potential side-effects may hinder its translation into clinical practice. On the other hand, pharmacological inhibition of neural receptors appears to be a reliable intervention that may be applied into clinical use [252,253]. Coinciding with the presumed tumor-facilitating role of β-AR signaling, epidemiological studies revealed that administration of β-blockers, such as propranolol, may reduce GI cancer risk [254,255,256]. Data obtained from in vitro and in vivo experiments also indicated that pharmacological inhibition of β-ARs retard GI cancer growth, invasion and chemoresistance [87,257]. These findings suggested a promising future of β-blockers in GI tumor prevention and therapy.

Other than β-blockers, nAChR inhibitors, such as α-bungarotoxin and mecamylamine, are candidate therapeutic agents for GI cancers [91,258]. While pre-clinical data have indicated apparent effect of these chemicals in blocking GI cancer malignancy, the in vivo efficacy and potential side-effects need to be addressed before they are considered for clinical use. In vitro studies also discovered anti-tumor effects of several other neurotransmitter pathway inhibitors in GI tumors. For instance, 5-HT receptor inhibitor vortioxetine may inhibit the viability, proliferation and invasion of gastric cancer cells [259]. Likewise, treatment with 5-HT2A inhibitor ketanserin and 5-HT3 ondansetron exacerbated ionizing radiation-induced cell death of colorectal cancer cells [260]. Recent studies also showed that administration with DAR antagonist pimozide reduced the growth and lymph node metastasis of colorectal cancer xenografts [261]. It should be noted that a significant proportion of these pharmacological agents have been under clinical use for a long period and can be quickly repurposed to cancer therapy. Cumulatively, these studies suggest that pharmacological blockade of neurotransmitter signaling pathways may serve as a valuable therapeutic strategy against GI cancer.

## 8. Conclusions

Mounting evidence has suggested that neural infiltration and neural signaling pathways play crucial roles in the development of GI tumors. Tumor-associated nerve fibers may fuel various aspects of tumor progression, including growth, metastasis, chemoresistance, angiogenesis and immune suppression. Neural receptor pathways, particularly those critically involved in the sympathetic and parasympathetic nervous systems such as β-AR and AChR pathways, are key players linking innervation and tumor progression. Pharmacological inhibitors of neurotransmitter pathways, such as β-blockers, have shown promising potential in the prevention and treatment of GI tumors. Herein, we reviewed recent progress in dissecting the role of tumor innervation in GI cancer progression. Given the fact that most of GI tissues are heavily innervated by enteric nerve fibers, it is conceivable that neural mechanisms play particularly important roles in GI tumor development. With the fast growth of new studies, the fascinating story of nerve and GI cancer partnership will continue.

## Figures and Tables

**Table 2:** The expression and roles of neurotrophin receptors in gastrointestinal cancer.

Receptors	Expression in Cancer/Mechanism	Downstream Effectors	Effects	Reference
p75^NTR^	Expressed in ESCC	Bmi-1	Self-renewal, proliferation, chemoresistance	[188,189,190]
	Downregulated in GC/DNA methylation	uPA, MMP-9, NF-κB	Impaired proliferation, invasion and metastasis	[126,191,192]
	Expressed in PC		Neural invasion, proliferation	[193,194]
	Downregulated in CRC/DNA methylation		Impaired proliferation, invasion and viability	[195]
	Downregulated in HCC		Impaired proliferation	[196,197]
TrkA	Upregulated in ESCC			[198]
	Expressed in GC			[199]
	Expressed in PC	PI3K/AKT	Chemoresistance	[200,201,202]
	Expressed in CRC	MAPK/ERK, MMP2, MMP9	metastasis	[182]
	Expressed in CC			[203]
	Upregulated in HCC/DNA demethlyation		Proliferation	[178]
TrkB	Expressed in ESCC		Chemoresistance	[204]
	Expressed in GC	Nrf2	lymph node metastasis, chemoresistance	[205,206,207]
	Expressed in PC		Invasion	[185,208]
	Upregulated in CRC	ERK	Proliferation, invasion, viability	[209,210,211,212]
	Upregulated in HCC/DNA demethlyation	RhoA, VEGF	Angiogenesis, proliferation, chemoresistance	[72,178,213]
TrkC	Expressed in GC			[199]
	Upregulated in PC			[149]
	Downregulated in CRC/DNA methylation		Impaired viability	[180,214,215]
	Upregulated in HCC/DNA demethlyation		Proliferation	[178]

ESCC, oesophageal squamous cell carcinoma; GC, gastric cancer; PC, pancreatic cancer; CRC, colorectal cancer; CC, cholangiocarcinoma; HCC, hepatocellular carcinoma.

## References

[1] Arnold M., Abnet C.C., Neale R.E., Vignat J., Giovannucci E.L., McGlynn K.A., Bray F. (2020). Global Burden of 5 Major Types of Gastrointestinal Cancer. Gastroenterology.

[2] Lu L., Mullins C.S., Schafmayer C., Zeissig S., Linnebacher M. (2021). A global assessment of recent trends in gastrointestinal cancer and lifestyle-associated risk factors. Cancer Commun..

[3] Silverman D.A., Martinez V.K., Dougherty P.M., Myers J.N., Calin G.A., Amit M. (2021). Cancer-Associated Neurogenesis and Nerve-Cancer Cross-talk. Cancer Res..

[4] Carney C.P., Jones L., Woolson R.F., Noyes R., Doebbeling B.N. (2003). Relationship between depression and pancreatic cancer in the general population. Psychosom. Med..

[5] Gregory E., Dugan R., David G., Song Y.H. (2020). The biology and engineered modeling strategies of cancer-nerve crosstalk. Biochim. Biophys. Acta Rev. Cancer.

[6] Kozlowska A., Kwiatkowski P., Oponowicz A., Majewski M., Kmiec Z., Godlewski J. (2016). Myenteric plexuses atrophy in the vicinity of colorectal cancer tissue is not caused by apoptosis or necrosis. Folia Histochem. Cytobiol..

[7] Wang B., Mou H., Liu M., Ran Z., Li X., Li J., Ou Y. (2021). Multiomics characteristics of neurogenesis-related gene are dysregulated in tumor immune microenvironment. Npj. Genom. Med..

[8] Yaman I., Cobanoglu D.A., Xie T., Ye Y., Amit M. (2022). Advances in understanding cancer-associated neurogenesis and its implications on the neuroimmune axis in cancer. Pharmacol. Ther..

[9] Griffin N., Faulkner S., Jobling P., Hondermarck H. (2018). Targeting neurotrophin signaling in cancer: The renaissance. Pharmacol. Res..

[10] Baraldi J.H., Martyn G.V., Shurin G.V., Shurin M.R. (2022). Tumor Innervation: History, Methodologies, and Significance. Cancers.

[11] Furness J.B., Callaghan B.P., Rivera L.R., Cho H.J. (2014). The enteric nervous system and gastrointestinal innervation: Integrated local and central control. Adv. Exp. Med. Biol..

[12] Gracie D.J., Hamlin P.J., Ford A.C. (2019). The influence of the brain-gut axis in inflammatory bowel disease and possible implications for treatment. Lancet Gastroenterol. Hepatol..

[13] Al Omran Y., Aziz Q. (2014). The brain-gut axis in health and disease. Adv. Exp. Med. Biol..

[14] Furness J.B. (2012). The enteric nervous system and neurogastroenterology. Nat. Rev. Gastroenterol. Hepatol..

[15] Spencer N.J., Hu H. (2020). Enteric nervous system: Sensory transduction, neural circuits and gastrointestinal motility. Nat. Rev. Gastroenterol. Hepatol..

[16] Shah E., Rezaie A., Riddle M., Pimentel M. (2014). Psychological disorders in gastrointestinal disease: Epiphenomenon, cause or consequence?. Ann. Gastroenterol..

[17] North C.S., Hong B.A., Alpers D.H. (2007). Relationship of functional gastrointestinal disorders and psychiatric disorders: Implications for treatment. World J. Gastroenterol..

[18] Levenstein S., Rosenstock S., Jacobsen R.K., Jorgensen T. (2015). Psychological stress increases risk for peptic ulcer, regardless of Helicobacter pylori infection or use of nonsteroidal anti-inflammatory drugs. Clin. Gastroenterol. Hepatol..

[19] Huang J., Valdimarsdottir U., Fall K., Ye W., Fang F. (2013). Pancreatic cancer risk after loss of a child: A register-based study in Sweden during 1991-2009. Am. J. Epidemiol..

[20] Kennedy B., Valdimarsdottir U., Sundstrom K., Sparen P., Lambe M., Fall K., Fang F. (2014). Loss of a parent and the risk of cancer in early life: A nationwide cohort study. Cancer Causes Control..

[21] Wang Y.H., Li J.Q., Shi J.F., Que J.Y., Liu J.J., Lappin J.M., Leung J., Ravindran A.V., Chen W.Q., Qiao Y.L. (2020). Depression and anxiety in relation to cancer incidence and mortality: A systematic review and meta-analysis of cohort studies. Mol. Psychiatry.

[22] Cao L., Liu X., Lin E.J., Wang C., Choi E.Y., Riban V., Lin B., During M.J. (2010). Environmental and genetic activation of a brain-adipocyte BDNF/leptin axis causes cancer remission and inhibition. Cell.

[23] Li G., Gan Y., Fan Y., Wu Y., Lin H., Song Y., Cai X., Yu X., Pan W., Yao M. (2015). Enriched environment inhibits mouse pancreatic cancer growth and down-regulates the expression of mitochondria-related genes in cancer cells. Sci. Rep..

[24] Zheng J., Cai W., Lu X., He W., Li D., Zhong H., Yang L., Li S., Li H., Rafee S. (2021). Chronic stress accelerates the process of gastric precancerous lesions in rats. J. Cancer.

[25] Ma S.R., Ma Q., Hao C.Q., Guan C.T., Li B.Y., Wang J.W., Li X.Q., Liu Z.K., Wei W.W. (2017). Analysis of psychological status and relevant factors of patients with esophageal and gastric cardia precancerous lesions in Linzhou of Henan. Zhonghua Yu Fang Yi Xue Za Zhi.

[26] Baritaki S., de Bree E., Chatzaki E., Pothoulakis C. (2019). Chronic Stress, Inflammation, and Colon Cancer: A CRH System-Driven Molecular Crosstalk. J. Clin. Med.

[27] Riley V. (1981). Psychoneuroendocrine influences on immunocompetence and neoplasia. Science.

[28] Kiecolt-Glaser J.K., Preacher K.J., MacCallum R.C., Atkinson C., Malarkey W.B., Glaser R. (2003). Chronic stress and age-related increases in the proinflammatory cytokine IL-6. Proc. Natl. Acad. Sci. USA.

[29] Dentino A.N., Pieper C.F., Rao M.K., Currie M.S., Harris T., Blazer D.G., Cohen H.J. (1999). Association of interleukin-6 and other biologic variables with depression in older people living in the community. J. Am. Geriatr. Soc..

[30] Rohleder N. (2014). Stimulation of systemic low-grade inflammation by psychosocial stress. Psychosom Med..

[31] Lutgendorf S.K., Garand L., Buckwalter K.C., Reimer T.T., Hong S.Y., Lubaroff D.M. (1999). Life stress, mood disturbance, and elevated interleukin-6 in healthy older women. J. Gerontol. A Biol. Sci. Med. Sci..

[32] Miller E.S., Apple C.G., Kannan K.B., Funk Z.M., Plazas J.M., Efron P.A., Mohr A.M. (2019). Chronic stress induces persistent low-grade inflammation. Am. J. Surg..

[33] Hara M.R., Kovacs J.J., Whalen E.J., Rajagopal S., Strachan R.T., Grant W., Towers A.J., Williams B., Lam C.M., Xiao K. (2011). A stress response pathway regulates DNA damage through beta2-adrenoreceptors and beta-arrestin-1. Nature.

[34] Feng Z., Liu L., Zhang C., Zheng T., Wang J., Lin M., Zhao Y., Wang X., Levine A.J., Hu W. (2012). Chronic restraint stress attenuates p53 function and promotes tumorigenesis. Proc. Natl. Acad. Sci. USA.

[35] Oertel H. (1928). Innervation and Tumour Growth: A Preliminary Report. Can. Med. Assoc. J..

[36] Shapiro D.M., Warren S. (1949). Cancer innervation. Cancer Res..

[37] Albo D., Akay C.L., Marshall C.L., Wilks J.A., Verstovsek G., Liu H., Agarwal N., Berger D.H., Ayala G.E. (2011). Neurogenesis in colorectal cancer is a marker of aggressive tumor behavior and poor outcomes. Cancer.

[38] Jeong S., Zheng B., Wang H., Xia Q., Chen L. (2018). Nervous system and primary liver cancer. Biochim. Biophys. Acta Rev. Cancer.

[39] Zhang L., Guo L., Tao M., Fu W., Xiu D. (2016). Parasympathetic neurogenesis is strongly associated with tumor budding and correlates with an adverse prognosis in pancreatic ductal adenocarcinoma. Chin. J. Cancer Res..

[40] Griffin N., Rowe C.W., Gao F., Jobling P., Wills V., Walker M.M., Faulkner S., Hondermarck H. (2020). Clinicopathological Significance of Nerves in Esophageal Cancer. Am. J. Pathol..

[41] Lu R., Fan C., Shangguan W., Liu Y., Li Y., Shang Y., Yin D., Zhang S., Huang Q., Li X. (2017). Neurons generated from carcinoma stem cells support cancer progression. Signal. Transduct. Target. Ther..

[42] Mauffrey P., Tchitchek N., Barroca V., Bemelmans A.P., Firlej V., Allory Y., Romeo P.H., Magnon C. (2019). Progenitors from the central nervous system drive neurogenesis in cancer. Nature.

[43] Chen S.H., Zhang B.Y., Zhou B., Zhu C.Z., Sun L.Q., Feng Y.J. (2019). Perineural invasion of cancer: A complex crosstalk between cells and molecules in the perineural niche. Am. J. Cancer Res..

[44] Deng J., You Q., Gao Y., Yu Q., Zhao P., Zheng Y., Fang W., Xu N., Teng L. (2014). Prognostic value of perineural invasion in gastric cancer: A systematic review and meta-analysis. PLoS ONE.

[45] Zeng Q., Cheng Y., Zhu Q., Yu Z., Wu X., Huang K., Zhou M., Han S., Zhang Q. (2008). The relationship between overexpression of glial cell-derived neurotrophic factor and its RET receptor with progression and prognosis of human pancreatic cancer. J. Int. Med. Res..

[46] Ceyhan G.O., Schafer K.H., Kerscher A.G., Rauch U., Demir I.E., Kadihasanoglu M., Bohm C., Muller M.W., Buchler M.W., Giese N.A. (2010). Nerve growth factor and artemin are paracrine mediators of pancreatic neuropathy in pancreatic adenocarcinoma. Ann. Surg..

[47] Hayakawa Y., Sakitani K., Konishi M., Asfaha S., Niikura R., Tomita H., Renz B.W., Tailor Y., Macchini M., Middelhoff M. (2017). Nerve Growth Factor Promotes Gastric Tumorigenesis through Aberrant Cholinergic Signaling. Cancer Cell.

[48] Arese M., Bussolino F., Pergolizzi M., Bizzozero L., Pascal D. (2018). Tumor progression: The neuronal input. Ann. Transl. Med..

[49] Schledwitz A., Xie G., Raufman J.P. (2021). Exploiting unique features of the gut-brain interface to combat gastrointestinal cancer. J. Clin Investig..

[50] Rademakers G., Vaes N., Schonkeren S., Koch A., Sharkey K.A., Melotte V. (2017). The role of enteric neurons in the development and progression of colorectal cancer. Biochim. Biophys. Acta Rev. Cancer.

[51] Cervantes-Villagrana R.D., Albores-Garcia D., Cervantes-Villagrana A.R., Garcia-Acevez S.J. (2020). Tumor-induced neurogenesis and immune evasion as targets of innovative anti-cancer therapies. Signal. Transduct. Target. Ther..

[52] Cavel O., Shomron O., Shabtay A., Vital J., Trejo-Leider L., Weizman N., Krelin Y., Fong Y., Wong R.J., Amit M. (2012). Endoneurial macrophages induce perineural invasion of pancreatic cancer cells by secretion of GDNF and activation of RET tyrosine kinase receptor. Cancer Res..

[53] Jensen K.J., Alpini G., Glaser S. (2013). Hepatic nervous system and neurobiology of the liver. Compr. Physiol..

[54] Wachsmuth H.R., Weninger S.N., Duca F.A. (2022). Role of the gut-brain axis in energy and glucose metabolism. Exp. Mol. Med..

[55] Tan X., Sivakumar S., Bednarsch J., Wiltberger G., Kather J.N., Niehues J., de Vos-Geelen J., Valkenburg-van Iersel L., Kintsler S., Roeth A. (2021). Nerve fibers in the tumor microenvironment in neurotropic cancer-pancreatic cancer and cholangiocarcinoma. Oncogene.

[56] Schonkeren S.L., Thijssen M.S., Vaes N., Boesmans W., Melotte V. (2021). The Emerging Role of Nerves and Glia in Colorectal Cancer. Cancers.

[57] Bednarsch J., Kather J., Tan X., Sivakumar S., Cacchi C., Wiltberger G., Czigany Z., Ulmer F., Neumann U.P., Heij L.R. (2021). Nerve Fibers in the Tumor Microenvironment as a Novel Biomarker for Oncological Outcome in Patients Undergoing Surgery for Perihilar Cholangiocarcinoma. Liver Cancer.

[58] Ferdoushi A., Griffin N., Marsland M., Xu X., Faulkner S., Gao F., Liu H., King S.J., Denham J.W., van Helden D.F. (2021). Tumor innervation and clinical outcome in pancreatic cancer. Sci. Rep..

[59] Gasparini G., Pellegatta M., Crippa S., Lena M.S., Belfiori G., Doglioni C., Taveggia C., Falconi M. (2019). Nerves and Pancreatic Cancer: New Insights into a Dangerous Relationship. Cancers.

[60] Terada T., Matsunaga Y. (2001). S-100-positive nerve fibers in hepatocellular carcinoma and intrahepatic cholangiocarcinoma: An immunohistochemical study. Pathol. Int..

[61] Zhang L., Yang L., Jiang S., Yu M. (2022). Nerve Dependence in Colorectal Cancer. Front. Cell Dev. Biol..

[62] Bednarsch J., Tan X., Czigany Z., Wiltberger G., Buelow R.D., Boor P., Lang S.A., Ulmer T.F., Neumann U.P., Heij L.R. (2022). Limitations of Nerve Fiber Density as a Prognostic Marker in Predicting Oncological Outcomes in Hepatocellular Carcinoma. Cancers.

[63] Holland A.M., Bon-Frauches A.C., Keszthelyi D., Melotte V., Boesmans W. (2021). The enteric nervous system in gastrointestinal disease etiology. Cell Mol. Life Sci..

[64] Berthoud H.R., Powley T.L. (1991). Morphology and distribution of efferent vagal innervation of rat pancreas as revealed with anterograde transport of Dil. Brain Res..

[65] Mizuno K., Ueno Y. (2017). Autonomic Nervous System and the Liver. Hepatol. Res..

[66] Zhao Y., Wang X., Wang T., Hu X., Hui X., Yan M., Gao Q., Chen T., Li J., Yao M. (2011). Acetylcholinesterase, a key prognostic predictor for hepatocellular carcinoma, suppresses cell growth and induces chemosensitization. Hepatology.

[67] Li J., Yang X.M., Wang Y.H., Feng M.X., Liu X.J., Zhang Y.L., Huang S., Wu Z., Xue F., Qin W.X. (2014). Monoamine oxidase A suppresses hepatocellular carcinoma metastasis by inhibiting the adrenergic system and its transactivation of EGFR signaling. J. Hepatol..

[68] Deborde S., Yu Y., Marcadis A., Chen C.H., Fan N., Bakst R.L., Wong R.J. (2018). An In Vivo Murine Sciatic Nerve Model of Perineural Invasion. J. Vis. Exp..

[69] Gohrig A., Detjen K.M., Hilfenhaus G., Korner J.L., Welzel M., Arsenic R., Schmuck R., Bahra M., Wu J.Y., Wiedenmann B. (2014). Axon guidance factor SLIT2 inhibits neural invasion and metastasis in pancreatic cancer. Cancer Res..

[70] Liu Z.S., Wang Y., Li Q., Zhang S.L., Shi Y.R. (2012). [In vitro interaction of human pancreatic cancer cells and rat dorsal root ganglia: A co-culture model]. Zhonghua Zhong Liu Za Zhi.

[71] Wu F.Q., Fang T., Yu L.X., Lv G.S., Lv H.W., Liang D., Li T., Wang C.Z., Tan Y.X., Ding J. (2016). ADRB2 signaling promotes HCC progression and sorafenib resistance by inhibiting autophagic degradation of HIF1alpha. J. Hepatol..

[72] Hajiasgharzadeh K., Somi M.H., Mansoori B., Khaze Shahgoli V., Derakhshani A., Mokhtarzadeh A., Shanehbandi D., Baradaran B. (2020). Small interfering RNA targeting alpha7 nicotinic acetylcholine receptor sensitizes hepatocellular carcinoma cells to sorafenib. Life Sci..

[73] Chen W.Y., Huang C.Y., Cheng W.L., Hung C.S., Huang M.T., Tai C.J., Liu Y.N., Chen C.L., Chang Y.J. (2015). Alpha 7-nicotinic acetylcholine receptor mediates the sensitivity of gastric cancer cells to 5-fluorouracil. Tumour Biol..

[74] Chen S., Kang X., Liu G., Zhang B., Hu X., Feng Y. (2019). Alpha7-Nicotinic Acetylcholine Receptor Promotes Cholangiocarcinoma Progression and Epithelial-Mesenchymal Transition Process. Dig. Dis. Sci..

[75] Lu Y., Zhang Y., Zhao H., Li Q., Liu Y., Zuo Y., Xu Q., Zuo H., Li Y. (2022). Chronic stress model simulated by salbutamol promotes tumorigenesis of gastric cancer cells through beta2-AR/ERK/EMT pathway. J. Cancer.

[76] Liu J., Qu L., Wan C., Xiao M., Ni W., Jiang F., Fan Y., Lu C., Ni R. (2020). A novel beta2-AR/YB-1/beta-catenin axis mediates chronic stress-associated metastasis in hepatocellular carcinoma. Oncogenesis.

[77] Gysler S.M., Drapkin R. (2021). Tumor innervation: Peripheral nerves take control of the tumor microenvironment. J. Clin. Investig..

[78] Reiter E., Ahn S., Shukla A.K., Lefkowitz R.J. (2012). Molecular mechanism of beta-arrestin-biased agonism at seven-transmembrane receptors. Annu. Rev. Pharmacol. Toxicol..

[79] Rains S.L., Amaya C.N., Bryan B.A. (2017). Beta-adrenergic receptors are expressed across diverse cancers. Oncoscience.

[80] Krizanova O., Babula P., Pacak K. (2016). Stress, catecholaminergic system and cancer. Stress.

[81] Perez-Sayans M., Somoza-Martin J.M., Barros-Angueira F., Diz P.G., Gandara Rey J.M., Garcia-Garcia A. (2010). Beta-adrenergic receptors in cancer: Therapeutic implications. Oncol. Res..

[82] Renz B.W., Takahashi R., Tanaka T., Macchini M., Hayakawa Y., Dantes Z., Maurer H.C., Chen X., Jiang Z., Westphalen C.B. (2018). Beta2 Adrenergic-Neurotrophin Feedforward Loop Promotes Pancreatic Cancer. Cancer Cell.

[83] Zhang D., Lei J., Ma J., Chen X., Sheng L., Jiang Z., Nan L., Xu Q., Duan W., Wang Z. (2016). Beta2-adrenogenic signaling regulates NNK-induced pancreatic cancer progression via upregulation of HIF-1alpha. Oncotarget.

[84] Shin V.Y., Wu W.K., Chu K.M., Koo M.W., Wong H.P., Lam E.K., Tai E.K., Cho C.H. (2007). Functional role of beta-adrenergic receptors in the mitogenic action of nicotine on gastric cancer cells. Toxicol. Sci..

[85] Shan T., Ma J., Ma Q., Guo K., Guo J., Li X., Li W., Liu J., Huang C., Wang F. (2013). Beta2-AR-HIF-1alpha: A novel regulatory axis for stress-induced pancreatic tumor growth and angiogenesis. Curr. Mol. Med..

[86] Chin C.C., Li J.M., Lee K.F., Huang Y.C., Wang K.C., Lai H.C., Cheng C.C., Kuo Y.H., Shi C.S. (2016). Selective beta2-AR Blockage Suppresses Colorectal Cancer Growth Through Regulation of EGFR-Akt/ERK1/2 Signaling, G1-Phase Arrest, and Apoptosis. J. Cell Physiol..

[87] Wan C., Gong C., Zhang H., Hua L., Li X., Chen X., Chen Y., Ding X., He S., Cao W. (2016). Beta2-adrenergic receptor signaling promotes pancreatic ductal adenocarcinoma (PDAC) progression through facilitating PCBP2-dependent c-myc expression. Cancer Lett..

[88] Hajiasgharzadeh K., Somi M.H., Sadigh-Eteghad S., Mokhtarzadeh A., Shanehbandi D., Mansoori B., Mohammadi A., Doustvandi M.A., Baradaran B. (2020). The dual role of alpha7 nicotinic acetylcholine receptor in inflammation-associated gastrointestinal cancers. Heliyon.

[89] Li L.F., Chan R.L., Lu L., Shen J., Zhang L., Wu W.K., Wang L., Hu T., Li M.X., Cho C.H. (2014). Cigarette smoking and gastrointestinal diseases: The causal relationship and underlying molecular mechanisms (review). Int. J. Mol. Med..

[90] Kabbani N., Nordman J.C., Corgiat B.A., Veltri D.P., Shehu A., Seymour V.A., Adams D.J. (2013). Are nicotinic acetylcholine receptors coupled to G proteins?. Bioessays.

[91] Momi N., Ponnusamy M.P., Kaur S., Rachagani S., Kunigal S.S., Chellappan S., Ouellette M.M., Batra S.K. (2013). Nicotine/cigarette smoke promotes metastasis of pancreatic cancer through alpha7nAChR-mediated MUC4 upregulation. Oncogene.

[92] Schaal C., Padmanabhan J., Chellappan S. (2015). The Role of nAChR and Calcium Signaling in Pancreatic Cancer Initiation and Progression. Cancers.

[93] Wan C., Wu M., Zhang S., Chen Y., Lu C. (2018). Alpha7nAChR-mediated recruitment of PP1gamma promotes TRAF6/NF-kappaB cascade to facilitate the progression of Hepatocellular Carcinoma. Mol. Carcinog..

[94] Takahashi T., Ohnishi H., Sugiura Y., Honda K., Suematsu M., Kawasaki T., Deguchi T., Fujii T., Orihashi K., Hippo Y. (2014). Non-neuronal acetylcholine as an endogenous regulator of proliferation and differentiation of Lgr5-positive stem cells in mice. FEBS J..

[95] Middelhoff M., Nienhuser H., Valenti G., Maurer H.C., Hayakawa Y., Takahashi R., Kim W., Jiang Z., Malagola E., Cuti K. (2020). Prox1-positive cells monitor and sustain the murine intestinal epithelial cholinergic niche. Nat. Commun.

[96] Raufman J.P., Samimi R., Shah N., Khurana S., Shant J., Drachenberg C., Xie G., Wess J., Cheng K. (2008). Genetic ablation of M3 muscarinic receptors attenuates murine colon epithelial cell proliferation and neoplasia. Cancer Res..

[97] Kodaira M., Kajimura M., Takeuchi K., Lin S., Hanai H., Kaneko E. (1999). Functional muscarinic m3 receptor expressed in gastric cancer cells stimulates tyrosine phosphorylation and MAP kinase. J. Gastroenterol..

[98] Ukegawa J.I., Takeuchi Y., Kusayanagi S., Mitamura K. (2003). Growth-promoting effect of muscarinic acetylcholine receptors in colon cancer cells. J. Cancer Res. Clin. Oncol..

[99] Park Y.S., Cho N.J. (2012). EGFR and PKC are involved in the activation of ERK1/2 and p90 RSK and the subsequent proliferation of SNU-407 colon cancer cells by muscarinic acetylcholine receptors. Mol. Cell Biochem..

[100] Yu H., Xia H., Tang Q., Xu H., Wei G., Chen Y., Dai X., Gong Q., Bi F. (2017). Acetylcholine acts through M3 muscarinic receptor to activate the EGFR signaling and promotes gastric cancer cell proliferation. Sci. Rep..

[101] Renz B.W., Tanaka T., Sunagawa M., Takahashi R., Jiang Z., Macchini M., Dantes Z., Valenti G., White R.A., Middelhoff M.A. (2018). Cholinergic Signaling via Muscarinic Receptors Directly and Indirectly Suppresses Pancreatic Tumorigenesis and Cancer Stemness. Cancer Discov..

[102] Pfitzinger P.L., Fangmann L., Wang K., Demir E., Gurlevik E., Fleischmann-Mundt B., Brooks J., D’Haese J.G., Teller S., Hecker A. (2020). Indirect cholinergic activation slows down pancreatic cancer growth and tumor-associated inflammation. J. Exp. Clin. Cancer Res..

[103] Hayashi S., Hamada T., Zaidi S.F., Oshiro M., Lee J., Yamamoto T., Ishii Y., Sasahara M., Kadowaki M. (2014). Nicotine suppresses acute colitis and colonic tumorigenesis associated with chronic colitis in mice. Am. J. Physiol. Gastrointest. Liver Physiol..

[104] Xiang T., Yu F., Fei R., Qian J., Chen W. (2016). CHRNA7 inhibits cell invasion and metastasis of LoVo human colorectal cancer cells through PI3K/Akt signaling. Oncol. Rep..

[105] Udupa S., Nguyen S., Hoang G., Nguyen T., Quinones A., Pham K., Asaka R., Nguyen K., Zhang C., Elgogary A. (2019). Upregulation of the Glutaminase II Pathway Contributes to Glutamate Production upon Glutaminase 1 Inhibition in Pancreatic Cancer. Proteomics.

[106] Stepulak A., Rola R., Polberg K., Ikonomidou C. (2014). Glutamate and its receptors in cancer. J. Neural Transm.

[107] Stepulak A., Luksch H., Gebhardt C., Uckermann O., Marzahn J., Sifringer M., Rzeski W., Staufner C., Brocke K.S., Turski L. (2009). Expression of glutamate receptor subunits in human cancers. Histochem. Cell Biol..

[108] Herner A., Sauliunaite D., Michalski C.W., Erkan M., De Oliveira T., Abiatari I., Kong B., Esposito I., Friess H., Kleeff J. (2011). Glutamate increases pancreatic cancer cell invasion and migration via AMPA receptor activation and Kras-MAPK signaling. Int. J. Cancer.

[109] Chang H.J., Yoo B.C., Lim S.B., Jeong S.Y., Kim W.H., Park J.G. (2005). Metabotropic glutamate receptor 4 expression in colorectal carcinoma and its prognostic significance. Clin. Cancer Res..

[110] Ferguson H.J., Wragg J.W., Ward S., Heath V.L., Ismail T., Bicknell R. (2016). Glutamate dependent NMDA receptor 2D is a novel angiogenic tumour endothelial marker in colorectal cancer. Oncotarget.

[111] Hu J., Duan W., Liu Y. (2020). Ketamine inhibits aerobic glycolysis in colorectal cancer cells by blocking the NMDA receptor-CaMK II-c-Myc pathway. Clin. Exp. Pharmacol. Physiol..

[112] Yan Z., Li P., Xue Y., Tian H., Zhou T., Zhang G. (2021). Glutamate receptor, ionotropic, Nmethyl Daspartateassociated protein 1 promotes colorectal cancer cell proliferation and metastasis, and is negatively regulated by miR2963p. Mol. Med. Rep..

[113] Watanabe K., Kanno T., Oshima T., Miwa H., Tashiro C., Nishizaki T. (2008). The NMDA receptor NR2A subunit regulates proliferation of MKN45 human gastric cancer cells. Biochem. Biophys. Res. Commun..

[114] Xu D.H., Li Q., Hu H., Ni B., Liu X., Huang C., Zhang Z.Z., Zhao G. (2018). Transmembrane protein GRINA modulates aerobic glycolysis and promotes tumor progression in gastric cancer. J. Exp. Clin. Cancer Res..

[115] Li L., Hanahan D. (2013). Hijacking the neuronal NMDAR signaling circuit to promote tumor growth and invasion. Cell.

[116] Eisenhofer G., Aneman A., Friberg P., Hooper D., Fandriks L., Lonroth H., Hunyady B., Mezey E. (1997). Substantial production of dopamine in the human gastrointestinal tract. J. Clin. Endocrinol. Metab.

[117] Mawe G.M., Hoffman J.M. (2013). Serotonin signalling in the gut—Functions, dysfunctions and therapeutic targets. Nat. Rev. Gastroenterol. Hepatol..

[118] Beaulieu J.M., Gainetdinov R.R. (2011). The physiology, signaling, and pharmacology of dopamine receptors. Pharmacol. Rev..

[119] Yan Y., Pan J., Chen Y., Xing W., Li Q., Wang D., Zhou X., Xie J., Miao C., Yuan Y. (2020). Increased dopamine and its receptor dopamine receptor D1 promote tumor growth in human hepatocellular carcinoma. Cancer Commun.

[120] Mu J., Huang W., Tan Z., Li M., Zhang L., Ding Q., Wu X., Lu J., Liu Y., Dong Q. (2017). Dopamine receptor D2 is correlated with gastric cancer prognosis. Oncol. Lett..

[121] Jandaghi P., Najafabadi H.S., Bauer A.S., Papadakis A.I., Fassan M., Hall A., Monast A., von Knebel Doeberitz M., Neoptolemos J.P., Costello E. (2016). Expression of DRD2 Is Increased in Human Pancreatic Ductal Adenocarcinoma and Inhibitors Slow Tumor Growth in Mice. Gastroenterology.

[122] Li T., Fu B., Zhang X., Zhou Y., Yang M., Cao M., Chen Y., Tan Y., Hu R. (2021). Overproduction of Gastrointestinal 5-HT Promotes Colitis-Associated Colorectal Cancer Progression via Enhancing NLRP3 Inflammasome Activation. Cancer Immunol. Res..

[123] Jiang S.H., Li J., Dong F.Y., Yang J.Y., Liu D.J., Yang X.M., Wang Y.H., Yang M.W., Fu X.L., Zhang X.X. (2017). Increased Serotonin Signaling Contributes to the Warburg Effect in Pancreatic Tumor Cells Under Metabolic Stress and Promotes Growth of Pancreatic Tumors in Mice. Gastroenterology.

[124] Gurbuz N., Ashour A.A., Alpay S.N., Ozpolat B. (2014). Down-regulation of 5-HT1B and 5-HT1D receptors inhibits proliferation, clonogenicity and invasion of human pancreatic cancer cells. PLoS ONE.

[125] Liu X., Wu W.K., Yu L., Sung J.J., Srivastava G., Zhang S.T., Cho C.H. (2008). Epinephrine stimulates esophageal squamous-cell carcinoma cell proliferation via beta-adrenoceptor-dependent transactivation of extracellular signal-regulated kinase/cyclooxygenase-2 pathway. J. Cell Biochem..

[126] Shi M., Liu D., Duan H., Han C., Wei B., Qian L., Chen C., Guo L., Hu M., Yu M. (2010). Catecholamine up-regulates MMP-7 expression by activating AP-1 and STAT3 in gastric cancer. Mol. Cancer.

[127] Hu H.T., Ma Q.Y., Zhang D., Shen S.G., Han L., Ma Y.D., Li R.F., Xie K.P. (2010). HIF-1alpha links beta-adrenoceptor agonists and pancreatic cancer cells under normoxic condition. Acta Pharmacol. Sin..

[128] Koh M., Takahashi T., Kurokawa Y., Kobayashi T., Saito T., Ishida T., Serada S., Fujimoto M., Naka T., Wada N. (2021). Propranolol suppresses gastric cancer cell growth by regulating proliferation and apoptosis. Gastric. Cancer.

[129] Takahashi K., Kaira K., Shimizu A., Sato T., Takahashi N., Ogawa H., Yoshinari D., Yokobori T., Asao T., Takeyoshi I. (2016). Clinical significance of beta2-adrenergic receptor expression in patients with surgically resected gastric adenocarcinoma. Tumour Biol..

[130] Xiao M.B., Jin D.D., Jiao Y.J., Ni W.K., Liu J.X., Qu L.S., Lu C.H., Ni R.Z., Jiang F., Chen W.C. (2018). Beta2-AR regulates the expression of AKR1B1 in human pancreatic cancer cells and promotes their proliferation via the ERK1/2 pathway. Mol. Biol. Rep..

[131] Gong C., Hu B., Chen H., Zhu J., Nie J., Hua L., Chen L., Fang Y., Hang C., Lu Y. (2022). Beta2-adrenergic receptor drives the metastasis and invasion of pancreatic ductal adenocarcinoma through activating Cdc42 signaling pathway. J. Mol. Histol..

[132] Ogawa H., Kaira K., Motegi Y., Yokobori T., Takada T., Kato R., Osone K., Takahashi R., Suga K., Ozawa N. (2020). Prognostic significance of beta2-adrenergic receptor expression in patients with surgically resected colorectal cancer. Int. J. Clin. Oncol..

[133] Chen D., Xing W., Hong J., Wang M., Huang Y., Zhu C., Yuan Y., Zeng W. (2012). The beta2-adrenergic receptor is a potential prognostic biomarker for human hepatocellular carcinoma after curative resection. Ann. Surg. Oncol..

[134] Perrone M.G., Notarnicola M., Caruso M.G., Tutino V., Scilimati A. (2008). Upregulation of beta3-adrenergic receptor mRNA in human colon cancer: A preliminary study. Oncology.

[135] Zhao Y., Zhou W., Xue L., Zhang W., Zhan Q. (2014). Nicotine activates YAP1 through nAChRs mediated signaling in esophageal squamous cell cancer (ESCC). PLoS ONE.

[136] Jia Y., Sun H., Wu H., Zhang H., Zhang X., Xiao D., Ma X., Wang Y. (2016). Nicotine Inhibits Cisplatin-Induced Apoptosis via Regulating alpha5-nAChR/AKT Signaling in Human Gastric Cancer Cells. PLoS ONE.

[137] Wang M., Li Y., Xiao Y., Yang M., Chen J., Jian Y., Chen X., Shi D., Ouyang Y., Kong L. (2021). Nicotine-mediated OTUD3 downregulation inhibits VEGF-C mRNA decay to promote lymphatic metastasis of human esophageal cancer. Nat. Commun..

[138] Lien Y.C., Wang W., Kuo L.J., Liu J.J., Wei P.L., Ho Y.S., Ting W.C., Wu C.H., Chang Y.J. (2011). Nicotine promotes cell migration through alpha7 nicotinic acetylcholine receptor in gastric cancer cells. Ann. Surg. Oncol..

[139] Wang W., Chin-Sheng H., Kuo L.J., Wei P.L., Lien Y.C., Lin F.Y., Liu H.H., Ho Y.S., Wu C.H., Chang Y.J. (2012). NNK enhances cell migration through alpha7-nicotinic acetylcholine receptor accompanied by increased of fibronectin expression in gastric cancer. Ann. Surg. Oncol..

[140] Nimmakayala R.K., Seshacharyulu P., Lakshmanan I., Rachagani S., Chugh S., Karmakar S., Rauth S., Vengoji R., Atri P., Talmon G.A. (2018). Cigarette Smoke Induces Stem Cell Features of Pancreatic Cancer Cells via PAF1. Gastroenterology.

[141] Ye Y.N., Liu E.S., Shin V.Y., Wu W.K., Cho C.H. (2004). The modulating role of nuclear factor-kappaB in the action of alpha7-nicotinic acetylcholine receptor and cross-talk between 5-lipoxygenase and cyclooxygenase-2 in colon cancer growth induced by 4-(N-methyl-N-nitrosamino)-1-(3-pyridyl)-1-butanone. J. Pharmacol. Exp. Ther..

[142] Wei P.L., Kuo L.J., Huang M.T., Ting W.C., Ho Y.S., Wang W., An J., Chang Y.J. (2011). Nicotine enhances colon cancer cell migration by induction of fibronectin. Ann. Surg. Oncol..

[143] Novotny A., Ryberg K., Heiman Ullmark J., Nilsson L., Khorram-Manesh A., Nordgren S., Delbro D.S., Nylund G. (2011). Is acetylcholine a signaling molecule for human colon cancer progression?. Scand J. Gastroenterol..

[144] Wei P.L., Chang Y.J., Ho Y.S., Lee C.H., Yang Y.Y., An J., Lin S.Y. (2009). Tobacco-specific carcinogen enhances colon cancer cell migration through alpha7-nicotinic acetylcholine receptor. Ann. Surg..

[145] Martinez A.K., Jensen K., Hall C., O’Brien A., Ehrlich L., White T., Meng F., Zhou T., Greene J., Bernuzzi F. (2017). Nicotine Promotes Cholangiocarcinoma Growth in Xenograft Mice. Am. J. Pathol..

[146] Zhang L., Wu L.L., Huan H.B., Wen X.D., Yang D.P., Chen D.F., Xia F. (2020). Activation of muscarinic acetylcholine receptor 1 promotes invasion of hepatocellular carcinoma by inducing epithelial-mesenchymal transition. Anticancer Drugs.

[147] Wang L., Xu J., Xia Y., Yin K., Li Z., Li B., Wang W., Xu H., Yang L., Xu Z. (2018). Muscarinic acetylcholine receptor 3 mediates vagus nerve-induced gastric cancer. Oncogenesis.

[148] Wang L., Zhi X., Zhang Q., Wei S., Li Z., Zhou J., Jiang J., Zhu Y., Yang L., Xu H. (2016). Muscarinic receptor M3 mediates cell proliferation induced by acetylcholine and contributes to apoptosis in gastric cancer. Tumour Biol..

[149] Zhang L., Xiu D., Zhan J., He X., Guo L., Wang J., Tao M., Fu W., Zhang H. (2016). High expression of muscarinic acetylcholine receptor 3 predicts poor prognosis in patients with pancreatic ductal adenocarcinoma. Onco Targets Ther..

[150] Hering N.A., Liu V., Kim R., Weixler B., Droeser R.A., Arndt M., Pozios I., Beyer K., Kreis M.E., Seeliger H. (2021). Blockage of Cholinergic Signaling via Muscarinic Acetylcholine Receptor 3 Inhibits Tumor Growth in Human Colorectal Adenocarcinoma. Cancers.

[151] Frucht H., Jensen R.T., Dexter D., Yang W.L., Xiao Y. (1999). Human colon cancer cell proliferation mediated by the M3 muscarinic cholinergic receptor. Clin. Cancer Res..

[152] Xie G., Cheng K., Shant J., Raufman J.P. (2009). Acetylcholine-induced activation of M3 muscarinic receptors stimulates robust matrix metalloproteinase gene expression in human colon cancer cells. Am. J. Physiol. Gastrointest. Liver Physiol..

[153] Cheng K., Zimniak P., Raufman J.P. (2003). Transactivation of the epidermal growth factor receptor mediates cholinergic agonist-induced proliferation of H508 human colon cancer cells. Cancer Res..

[154] Feng Y.J., Zhang B.Y., Yao R.Y., Lu Y. (2012). Muscarinic acetylcholine receptor M3 in proliferation and perineural invasion of cholangiocarcinoma cells. Hepatobiliary Pancreat Dis. Int..

[155] Vega-Benedetti A.F., Loi E., Moi L., Restivo A., Cabras F., Deidda S., Pretta A., Ziranu P., Orru S., Scartozzi M. (2022). Colorectal cancer promoter methylation alteration affects the expression of glutamate ionotropic receptor AMPA type subunit 4 alternative isoforms potentially relevant in colon tissue. Hum. Cell.

[156] North W.G., Liu F., Lin L.Z., Tian R., Akerman B. (2017). NMDA receptors are important regulators of pancreatic cancer and are potential targets for treatment. Clin. Pharmacol..

[157] Chen X., Wu Q., You L., Chen S., Zhu M., Miao C. (2017). Propofol attenuates pancreatic cancer malignant potential via inhibition of NMDA receptor. Eur. J. Pharmacol..

[158] Duan W., Hu J., Liu Y. (2019). Ketamine inhibits colorectal cancer cells malignant potential via blockage of NMDA receptor. Exp. Mol. Pathol..

[159] Stepanov Y.V., Golovynska I., Dziubenko N.V., Kuznietsova H.M., Petriv N., Skrypkina I., Golovynskyi S., Stepanova L.I., Stohnii Y., Garmanchuk L.V. (2022). NMDA receptor expression during cell transformation process at early stages of liver cancer in rodent models. Am. J. Physiol. Gastrointest. Liver Physiol..

[160] Wu C.S., Lu Y.J., Li H.P., Hsueh C., Lu C.Y., Leu Y.W., Liu H.P., Lin K.H., Hui-Ming Huang T., Chang Y.S. (2010). Glutamate receptor, ionotropic, kainate 2 silencing by DNA hypermethylation possesses tumor suppressor function in gastric cancer. Int. J. Cancer.

[161] Gong B., Li Y., Cheng Z., Wang P., Luo L., Huang H., Duan S., Liu F. (2017). GRIK3: A novel oncogenic protein related to tumor TNM stage, lymph node metastasis, and poor prognosis of GC. Tumour Biol..

[162] Ye Y., Li Y., Wei Y., Xu Y., Wang R., Fu Z., Zheng S., Zhou Q., Zhou Y., Chen R. (2018). Anticancer effect of HOTTIP regulates human pancreatic cancer via the metabotropic glutamate receptor 1 pathway. Oncol. Lett..

[163] Wang X., Xiao L., Yu H. (2018). Expression levels of long non-coding RNA HOXA distal transcript antisense RNA and metabotropic glutamate receptor 1 in pancreatic carcinoma, and their prognostic values. Oncol. Lett..

[164] Yoo B.C., Jeon E., Hong S.H., Shin Y.K., Chang H.J., Park J.G. (2004). Metabotropic glutamate receptor 4-mediated 5-Fluorouracil resistance in a human colon cancer cell line. Clin. Cancer Res..

[165] Yang H.M., Hou T.Z., Zhang Y.N., Zhao S.D., Wu Y.L., Zhang H. (2022). Blocked metabotropic glutamate receptor 5 enhances chemosensitivity in hepatocellular carcinoma and attenuates chemotoxicity in the normal liver by regulating DNA damage. Cancer Gene Ther..

[166] Li L., Miyamoto M., Ebihara Y., Mega S., Takahashi R., Hase R., Kaneko H., Kadoya M., Itoh T., Shichinohe T. (2006). DRD2/DARPP-32 expression correlates with lymph node metastasis and tumor progression in patients with esophageal squamous cell carcinoma. World J. Surg..

[167] Su H., Xue Z., Feng Y., Xie Y., Deng B., Yao Y., Tian X., An Q., Yang L., Yao Q. (2019). N-arylpiperazine-containing compound (C2): An enhancer of sunitinib in the treatment of pancreatic cancer, involving D1DR activation. Toxicol. Appl. Pharmacol..

[168] Qian X., Zhang D., Cao Z., Ma H. (2021). Dopamine Pathway Mediated by DRD5 Facilitates Tumor Growth via Enhancing Warburg Effect in Esophageal Cancer. Front. Oncol..

[169] Leng Z.G., Lin S.J., Wu Z.R., Guo Y.H., Cai L., Shang H.B., Tang H., Xue Y.J., Lou M.Q., Zhao W. (2017). Activation of DRD5 (dopamine receptor D5) inhibits tumor growth by autophagic cell death. Autophagy.

[170] Lu M., Li J., Luo Z., Zhang S., Xue S., Wang K., Shi Y., Zhang C., Chen H., Li Z. (2015). Roles of dopamine receptors and their antagonist thioridazine in hepatoma metastasis. Onco Targets Ther..

[171] Ataee R., Ajdary S., Zarrindast M., Rezayat M., Hayatbakhsh M.R. (2010). Anti-mitogenic and apoptotic effects of 5-HT1B receptor antagonist on HT29 colorectal cancer cell line. J. Cancer Res. Clin. Oncol..

[172] Ataee R., Ajdary S., Rezayat M., Shokrgozar M.A., Shahriari S., Zarrindast M.R. (2010). Study of 5HT3 and HT4 receptor expression in HT29 cell line and human colon adenocarcinoma tissues. Arch. Iran. Med..

[173] Nocito A., Dahm F., Jochum W., Jang J.H., Georgiev P., Bader M., Graf R., Clavien P.A. (2008). Serotonin regulates macrophage-mediated angiogenesis in a mouse model of colon cancer allografts. Cancer Res..

[174] Ataee R., Ajdary S., Zarrindast M., Rezayat M., Shokrgozar M.A., Ataee A. (2010). Y25130 hydrochloride, a selective 5HT3 receptor antagonist has potent antimitogenic and apoptotic effect on HT29 colorectal cancer cell line. Eur. J. Cancer Prev..

[175] Alpini G., Invernizzi P., Gaudio E., Venter J., Kopriva S., Bernuzzi F., Onori P., Franchitto A., Coufal M., Frampton G. (2008). Serotonin metabolism is dysregulated in cholangiocarcinoma, which has implications for tumor growth. Cancer Res..

[176] Soll C., Riener M.O., Oberkofler C.E., Hellerbrand C., Wild P.J., DeOliveira M.L., Clavien P.A. (2012). Expression of serotonin receptors in human hepatocellular cancer. Clin. Cancer Res..

[177] Blondy S., Christou N., David V., Verdier M., Jauberteau M.O., Mathonnet M., Perraud A. (2019). Neurotrophins and their involvement in digestive cancers. Cell Death Dis..

[178] Jin W., Lee J.J., Kim M.S., Son B.H., Cho Y.K., Kim H.P. (2011). DNA methylation-dependent regulation of TrkA, TrkB, and TrkC genes in human hepatocellular carcinoma. Biochem. Biophys. Res. Commun..

[179] Fujimoto M., Kitazawa R., Maeda S., Kitazawa S. (2005). Methylation adjacent to negatively regulating AP-1 site reactivates TrkA gene expression during cancer progression. Oncogene.

[180] Luo Y., Kaz A.M., Kanngurn S., Welsch P., Morris S.M., Wang J., Lutterbaugh J.D., Markowitz S.D., Grady W.M. (2013). NTRK3 is a potential tumor suppressor gene commonly inactivated by epigenetic mechanisms in colorectal cancer. PLoS Genet..

[181] Lin H., Huang H., Yu Y., Chen W., Zhang S., Zhang Y. (2021). Nerve growth factor regulates liver cancer cell polarity and motility. Mol. Med. Rep..

[182] Lei Y., He X., Huang H., He Y., Lan J., Yang J., Liu W., Zhang T. (2022). Nerve growth factor orchestrates NGAL and matrix metalloproteinases activity to promote colorectal cancer metastasis. Clin. Transl. Oncol..

[183] Jiang J., Bai J., Qin T., Wang Z., Han L. (2020). NGF from pancreatic stellate cells induces pancreatic cancer proliferation and invasion by PI3K/AKT/GSK signal pathway. J. Cell Mol. Med..

[184] Okugawa Y., Tanaka K., Inoue Y., Kawamura M., Kawamoto A., Hiro J., Saigusa S., Toiyama Y., Ohi M., Uchida K. (2013). Brain-derived neurotrophic factor/tropomyosin-related kinase B pathway in gastric cancer. Br. J. Cancer.

[185] Oyama Y., Nagao S., Na L., Yanai K., Umebayashi M., Nakamura K., Nagai S., Fujimura A., Yamasaki A., Nakayama K. (2021). TrkB/BDNF Signaling Could Be a New Therapeutic Target for Pancreatic Cancer. Anticancer Res..

[186] Kim M.S., Suh K.W., Hong S., Jin W. (2017). TrkC promotes colorectal cancer growth and metastasis. Oncotarget.

[187] Demir I.E., Tieftrunk E., Schorn S., Friess H., Ceyhan G.O. (2016). Nerve growth factor & TrkA as novel therapeutic targets in cancer. Biochim. Biophys. Acta.

[188] Okumura T., Tsunoda S., Mori Y., Ito T., Kikuchi K., Wang T.C., Yasumoto S., Shimada Y. (2006). The biological role of the low-affinity p75 neurotrophin receptor in esophageal squamous cell carcinoma. Clin. Cancer Res..

[189] Huang S.D., Yuan Y., Liu X.H., Gong D.J., Bai C.G., Wang F., Luo J.H., Xu Z.Y. (2009). Self-renewal and chemotherapy resistance of p75NTR positive cells in esophageal squamous cell carcinomas. BMC Cancer.

[190] Kojima H., Okumura T., Yamaguchi T., Miwa T., Shimada Y., Nagata T. (2017). Enhanced cancer stem cell properties of a mitotically quiescent subpopulation of p75NTR-positive cells in esophageal squamous cell carcinoma. Int. J. Oncol..

[191] Jin H., Pan Y., He L., Zhai H., Li X., Zhao L., Sun L., Liu J., Hong L., Song J. (2007). p75 neurotrophin receptor inhibits invasion and metastasis of gastric cancer. Mol. Cancer Res..

[192] Jin H., Wu Z., Tan B., Liu Z., Zu Z., Wu X., Bi Y., Hu X. (2022). Ibuprofen promotes p75 neurotrophin receptor expression through modifying promoter methylation and N6-methyladenosine-RNA-methylation in human gastric cancer cells. Bioengineered.

[193] Bapat A.A., Munoz R.M., Von Hoff D.D., Han H. (2016). Blocking Nerve Growth Factor Signaling Reduces the Neural Invasion Potential of Pancreatic Cancer Cells. PLoS ONE.

[194] Zhu Z., Kleeff J., Kayed H., Wang L., Korc M., Buchler M.W., Friess H. (2002). Nerve growth factor and enhancement of proliferation, invasion, and tumorigenicity of pancreatic cancer cells. Mol. Carcinog..

[195] Yang Z., Chen H., Huo L., Bai Y., Fan X., Ni B., Fang L., Hu J., Peng J., Wang L. (2015). Epigenetic inactivation and tumor-suppressor behavior of NGFR in human colorectal cancer. Mol. Cancer Res..

[196] Yuanlong H., Haifeng J., Xiaoyin Z., Jialin S., Jie L., Li Y., Huahong X., Jiugang S., Yanglin P., Kaichun W. (2008). The inhibitory effect of p75 neurotrophin receptor on growth of human hepatocellular carcinoma cells. Cancer Lett..

[197] Tokusashi Y., Asai K., Tamakawa S., Yamamoto M., Yoshie M., Yaginuma Y., Miyokawa N., Aoki T., Kino S., Kasai S. (2005). Expression of NGF in hepatocellular carcinoma cells with its receptors in non-tumor cell components. Int. J. Cancer.

[198] Griffin N., Gao F., Jobling P., Oldmeadow C., Wills V., Walker M.M., Faulkner S., Hondermarck H. (2021). The neurotrophic tyrosine kinase receptor 1 (TrkA) is overexpressed in oesophageal squamous cell carcinoma. Pathology.

[199] Kamiya A., Inokuchi M., Otsuki S., Sugita H., Kato K., Uetake H., Sugihara K., Takagi Y., Kojima K. (2016). Prognostic value of tropomyosin-related kinases A, B, and C in gastric cancer. Clin. Transl. Oncol..

[200] Schneider M.B., Standop J., Ulrich A., Wittel U., Friess H., Andren-Sandberg A., Pour P.M. (2001). Expression of nerve growth factors in pancreatic neural tissue and pancreatic cancer. J. Histochem. Cytochem..

[201] Dang C., Zhang Y., Ma Q., Shimahara Y. (2006). Expression of nerve growth factor receptors is correlated with progression and prognosis of human pancreatic cancer. J. Gastroenterol. Hepatol..

[202] Liu D., Zhang Y., Dang C., Ma Q., Lee W., Chen W. (2007). siRNA directed against TrkA sensitizes human pancreatic cancer cells to apoptosis induced by gemcitabine through an inactivation of PI3K/Akt-dependent pathway. Oncol. Rep..

[203] Yang X.Q., Xu Y.F., Guo S., Liu Y., Ning S.L., Lu X.F., Yang H., Chen Y.X. (2014). Clinical significance of nerve growth factor and tropomyosin-receptor-kinase signaling pathway in intrahepatic cholangiocarcinoma. World J. Gastroenterol..

[204] Tanaka K., Mohri Y., Nishioka J., Ohi M., Yokoe T., Miki C., Tonouchi H., Nobori T., Kusunoki M. (2009). Neurotrophic receptor, tropomyosin-related kinase B, as a chemoresistant marker in oesophageal cancer. Clin. Oncol..

[205] Tanaka K., Mohri Y., Nishioka J., Kobayashi M., Ohi M., Miki C., Tonouchi H., Nobori T., Kusunoki M. (2009). Neurotrophic receptor, tropomyosin-related kinase B as an independent prognostic marker in gastric cancer patients. J. Surg. Oncol..

[206] Zhao M.X., Ding S.G., Liu L.N., Wang Y., Zhang J., Zhang H.J., Zhang Y. (2012). [Predicative value of expression of TrkB and TRIM29 in biopsy tissues from preoperative gastroscopy in lymph node metastasis of gastric cancer]. Zhonghua Yi Xue Za Zhi.

[207] Jin Z., Lu Y., Wu X., Pan T., Yu Z., Hou J., Wu A., Li J., Yang Z., Li C. (2021). The cross-talk between tumor cells and activated fibroblasts mediated by lactate/BDNF/TrkB signaling promotes acquired resistance to anlotinib in human gastric cancer. Redox Biol..

[208] Sclabas G.M., Fujioka S., Schmidt C., Li Z., Frederick W.A., Yang W., Yokoi K., Evans D.B., Abbruzzese J.L., Hess K.R. (2005). Overexpression of tropomysin-related kinase B in metastatic human pancreatic cancer cells. Clin. Cancer Res..

[209] Yu Y., Zhang S., Wang X., Yang Z., Ou G. (2010). Overexpression of TrkB promotes the progression of colon cancer. APMIS.

[210] De Farias C.B., Heinen T.E., dos Santos R.P., Abujamra A.L., Schwartsmann G., Roesler R. (2012). BDNF/TrkB signaling protects HT-29 human colon cancer cells from EGFR inhibition. Biochem. Biophys. Res. Commun..

[211] Liu L., Li S.W., Yuan W., Tang J., Sang Y. (2021). Downregulation of SUN2 promotes metastasis of colon cancer by activating BDNF/TrkB signalling by interacting with SIRT1. J. Pathol..

[212] Park G.B., Choi S., Yoon Y.S., Kim D. (2021). TrkB/C-induced HOXC6 activation enhances the ADAM8-mediated metastasis of chemoresistant colon cancer cells. Mol. Med. Rep..

[213] Lam C.T., Yang Z.F., Lau C.K., Tam K.H., Fan S.T., Poon R.T. (2011). Brain-derived neurotrophic factor promotes tumorigenesis via induction of neovascularization: Implication in hepatocellular carcinoma. Clin. Cancer Res..

[214] Chen Z., Huang Z., Luo Y., Zou Q., Bai L., Tang G., Wang X., Cao G., Huang M., Xiang J. (2021). Genome-wide analysis identifies critical DNA methylations within NTRKs genes in colorectal cancer. J. Transl. Med..

[215] Genevois A.L., Ichim G., Coissieux M.M., Lambert M.P., Lavial F., Goldschneider D., Jarrosson-Wuilleme L., Lepinasse F., Gouysse G., Herceg Z. (2013). Dependence receptor TrkC is a putative colon cancer tumor suppressor. Proc. Natl. Acad. Sci. USA.

[216] Botticelli L., Micioni Di Bonaventura E., Ubaldi M., Ciccocioppo R., Cifani C., Micioni Di Bonaventura M.V. (2021). The Neural Network of Neuropeptide S (NPS): Implications in Food Intake and Gastrointestinal Functions. Pharmaceuticals.

[217] Arora S., Anubhuti (2006). Role of neuropeptides in appetite regulation and obesity--A review. Neuropeptides.

[218] Wei P., Keller C., Li L. (2020). Neuropeptides in gut-brain axis and their influence on host immunity and stress. Comput. Struct. Biotechnol. J..

[219] Stevenson L., Allen W.L., Turkington R., Jithesh P.V., Proutski I., Stewart G., Lenz H.J., Van Schaeybroeck S., Longley D.B., Johnston P.G. (2012). Identification of galanin and its receptor GalR1 as novel determinants of resistance to chemotherapy and potential biomarkers in colorectal cancer. Clin. Cancer Res..

[220] Nagayoshi K., Ueki T., Tashiro K., Mizuuchi Y., Manabe T., Araki H., Oda Y., Kuhara S., Tanaka M. (2015). Galanin plays an important role in cancer invasiveness and is associated with poor prognosis in stage II colorectal cancer. Oncol. Rep..

[221] Yoon D., Bae K., Lee M.K., Kim J.H., Yoon K.A. (2018). Galanin is an epigenetically silenced tumor suppressor gene in gastric cancer cells. PLoS ONE.

[222] Zhang L., Fang P., Chai C., Shao L., Mao H., Qiao D., Kong G., Dong X., Shi M., Zhang Z. (2019). Galanin expression is down-regulated in patients with gastric cancer. J. Int. Med. Res..

[223] Wang X., Jiao X., Meng Y., Chen H., Griffin N., Gao X., Shan F. (2018). Methionine enkephalin (MENK) inhibits human gastric cancer through regulating tumor associated macrophages (TAMs) and PI3K/AKT/mTOR signaling pathway inside cancer cells. Int. Immunopharmacol..

[224] Wang X., Tian J., Jiao X., Geng J., Wang R., Liu N., Gao X., Griffin N., Gao Y., Shan F. (2018). The novel mechanism of anticancer effect on gastric cancer through inducing G0/G1 cell cycle arrest and caspase-dependent apoptosis in vitro and in vivo by methionine enkephalin. Cancer Manag. Res..

[225] Li Y., Chen S., Li Z. (1998). Plasma neuropeptide Y (NPY) levels in patients with gastric and colorectal carcinomas. Zhonghua Zhong Liu Za Zhi.

[226] Jeppsson S., Srinivasan S., Chandrasekharan B. (2017). Neuropeptide Y (NPY) promotes inflammation-induced tumorigenesis by enhancing epithelial cell proliferation. Am. J. Physiol. Gastrointest. Liver Physiol..

[227] Demir I.E., Friess H., Ceyhan G.O. (2012). Nerve-cancer interactions in the stromal biology of pancreatic cancer. Front. Physiol..

[228] Hondermarck H., Jobling P. (2018). The Sympathetic Nervous System Drives Tumor Angiogenesis. Trends Cancer.

[229] Macklin K.D., Maus A.D., Pereira E.F., Albuquerque E.X., Conti-Fine B.M. (1998). Human vascular endothelial cells express functional nicotinic acetylcholine receptors. J. Pharmacol. Exp. Ther..

[230] Schuller H.M., Al-Wadei H.A., Ullah M.F., Plummer H.K. (2012). Regulation of pancreatic cancer by neuropsychological stress responses: A novel target for intervention. Carcinogenesis.

[231] Lu Y., Xu Q., Zuo Y., Liu L., Liu S., Chen L., Wang K., Lei Y., Zhao X., Li Y. (2017). Isoprenaline/beta2-AR activates Plexin-A1/VEGFR2 signals via VEGF secretion in gastric cancer cells to promote tumor angiogenesis. BMC Cancer.

[232] Chakroborty D., Sarkar C., Basu B., Dasgupta P.S., Basu S. (2009). Catecholamines regulate tumor angiogenesis. Cancer Res..

[233] Chakroborty D., Sarkar C., Mitra R.B., Banerjee S., Dasgupta P.S., Basu S. (2004). Depleted dopamine in gastric cancer tissues: Dopamine treatment retards growth of gastric cancer by inhibiting angiogenesis. Clin. Cancer Res..

[234] Basu S., Nagy J.A., Pal S., Vasile E., Eckelhoefer I.A., Bliss V.S., Manseau E.J., Dasgupta P.S., Dvorak H.F., Mukhopadhyay D. (2001). The neurotransmitter dopamine inhibits angiogenesis induced by vascular permeability factor/vascular endothelial growth factor. Nat. Med..

[235] Schiller M., Ben-Shaanan T.L., Rolls A. (2021). Neuronal regulation of immunity: Why, how and where?. Nat. Rev. Immunol..

[236] Wu J.J., Yang Y., Peng W.T., Sun J.C., Sun W.Y., Wei W. (2019). G protein-coupled receptor kinase 2 regulating beta2-adrenergic receptor signaling in M2-polarized macrophages contributes to hepatocellular carcinoma progression. Onco Targets Ther..

[237] Fei R., Zhang Y., Wang S., Xiang T., Chen W. (2017). Alpha7 nicotinic acetylcholine receptor in tumor-associated macrophages inhibits colorectal cancer metastasis through the JAK2/STAT3 signaling pathway. Oncol. Rep..

[238] Ganor Y., Levite M. (2014). The neurotransmitter glutamate and human T cells: Glutamate receptors and glutamate-induced direct and potent effects on normal human T cells, cancerous human leukemia and lymphoma T cells, and autoimmune human T cells. J. Neural Transm..

[239] Qiao G., Chen M., Mohammadpour H., MacDonald C.R., Bucsek M.J., Hylander B.L., Barbi J.J., Repasky E.A. (2021). Chronic Adrenergic Stress Contributes to Metabolic Dysfunction and an Exhausted Phenotype in T Cells in the Tumor Microenvironment. Cancer Immunol Res..

[240] Partecke L.I., Speerforck S., Kading A., Seubert F., Kuhn S., Lorenz E., Schwandke S., Sendler M., Kessler W., Trung D.N. (2016). Chronic stress increases experimental pancreatic cancer growth, reduces survival and can be antagonised by beta-adrenergic receptor blockade. Pancreatology.

[241] Yang M.W., Tao L.Y., Jiang Y.S., Yang J.Y., Huo Y.M., Liu D.J., Li J., Fu X.L., He R., Lin C. (2020). Perineural Invasion Reprograms the Immune Microenvironment through Cholinergic Signaling in Pancreatic Ductal Adenocarcinoma. Cancer Res..

[242] Tanaka A., Watanabe T., Okuno K., Yasutomi M. (1994). Perineural invasion as a predictor of recurrence of gastric cancer. Cancer.

[243] Knijn N., Mogk S.C., Teerenstra S., Simmer F., Nagtegaal I.D. (2016). Perineural Invasion is a Strong Prognostic Factor in Colorectal Cancer: A Systematic Review. Am. J. Surg. Pathol..

[244] Demir I.E., Ceyhan G.O., Liebl F., D’Haese J.G., Maak M., Friess H. (2010). Neural invasion in pancreatic cancer: The past, present and future. Cancers.

[245] Guo K., Ma Q., Li J., Wang Z., Shan T., Li W., Xu Q., Xie K. (2013). Interaction of the sympathetic nerve with pancreatic cancer cells promotes perineural invasion through the activation of STAT3 signaling. Mol. Cancer Ther..

[246] Li X., Ma G., Ma Q., Li W., Liu J., Han L., Duan W., Xu Q., Liu H., Wang Z. (2013). Neurotransmitter substance P mediates pancreatic cancer perineural invasion via NK-1R in cancer cells. Mol. Cancer Res..

[247] Shan T., Cui X., Li W., Lin W., Li Y., Chen X., Wu T. (2014). Novel regulatory program for norepinephrine-induced epithelial-mesenchymal transition in gastric adenocarcinoma cell lines. Cancer Sci..

[248] Zhao C.M., Hayakawa Y., Kodama Y., Muthupalani S., Westphalen C.B., Andersen G.T., Flatberg A., Johannessen H., Friedman R.A., Renz B.W. (2014). Denervation suppresses gastric tumorigenesis. Sci. Transl. Med..

[249] Saloman J.L., Albers K.M., Li D., Hartman D.J., Crawford H.C., Muha E.A., Rhim A.D., Davis B.M. (2016). Ablation of sensory neurons in a genetic model of pancreatic ductal adenocarcinoma slows initiation and progression of cancer. Proc. Natl. Acad. Sci. USA.

[250] Liu V., Dietrich A., Kasparek M.S., Benhaqi P., Schneider M.R., Schemann M., Seeliger H., Kreis M.E. (2015). Extrinsic intestinal denervation modulates tumor development in the small intestine of Apc(Min/+) mice. J. Exp. Clin. Cancer Res..

[251] Sadighparvar S., Darband S.G., Ghaderi-Pakdel F., Mihanfar A., Majidinia M. (2021). Parasympathetic, but not sympathetic denervation, suppressed colorectal cancer progression. Eur. J. Pharmacol..

[252] Hu Q., Liao P., Li W., Hu J., Chen C., Zhang Y., Wang Y., Chen L., Song K., Liu J. (2021). Clinical Use of Propranolol Reduces Biomarkers of Proliferation in Gastric Cancer. Front. Oncol..

[253] Hermawan A., Putri H., Utomo R.Y. (2020). Functional network analysis reveals potential repurposing of beta-blocker atenolol for pancreatic cancer therapy. Daru.

[254] Chang P.Y., Huang W.Y., Lin C.L., Huang T.C., Wu Y.Y., Chen J.H., Kao C.H. (2015). Propranolol Reduces Cancer Risk: A Population-Based Cohort Study. Medicine.

[255] Nkontchou G., Aout M., Mahmoudi A., Roulot D., Bourcier V., Grando-Lemaire V., Ganne-Carrie N., Trinchet J.C., Vicaut E., Beaugrand M. (2012). Effect of long-term propranolol treatment on hepatocellular carcinoma incidence in patients with HCV-associated cirrhosis. Cancer Prev. Res..

[256] Saad A., Goldstein J., Margalit O., Shacham-Shmueli E., Lawrence Y.R., Yang Y.X., Reiss K.A., Golan T., Mamtani R., Halpern N. (2020). Assessing the effects of beta-blockers on pancreatic cancer risk: A nested case-control study. Pharmacoepidemiol. Drug Saf..

[257] Zhang D., Ma Q.Y., Hu H.T., Zhang M. (2010). Beta2-adrenergic antagonists suppress pancreatic cancer cell invasion by inhibiting CREB, NFkappaB and AP-1. Cancer Biol. Ther..

[258] Shin V.Y., Jin H.C., Ng E.K., Yu J., Leung W.K., Cho C.H., Sung J.J. (2008). Nicotine and 4-(methylnitrosamino)-1-(3-pyridyl)-1-butanone induce cyclooxygenase-2 activity in human gastric cancer cells: Involvement of nicotinic acetylcholine receptor (nAChR) and beta-adrenergic receptor signaling pathways. Toxicol. Appl. Pharmacol..

[259] Lv G.B., Wang T.T., Zhu H.L., Wang H.K., Sun W., Zhao L.F. (2020). Vortioxetine induces apoptosis and autophagy of gastric cancer AGS cells via the PI3K/AKT pathway. FEBS Open Bio.

[260] Curtis J.J., Vo N.T.K., Seymour C.B., Mothersill C.E. (2020). 5-HT2A and 5-HT3 receptors contribute to the exacerbation of targeted and non-targeted effects of ionizing radiation-induced cell death in human colon carcinoma cells. Int. J. Radiat. Biol..

[261] Lee H., Shim S., Kong J.S., Kim M.J., Park S., Lee S.S., Kim A. (2021). Overexpression of dopamine receptor D2 promotes colorectal cancer progression by activating the beta-catenin/ZEB1 axis. Cancer Sci..

